# Benchmarking machine learning and molecular dynamics methods for the prediction of the thermophysical properties of organic liquids

**DOI:** 10.1039/d6dd00260a

**Published:** 2026-09-29

**Authors:** Egheosa Ogbomo, Fionn Carman, Daniele Dini, James P. Ewen

**Affiliations:** a Department of Mechanical Engineering, Imperial College London South Kensington Campus London SW7 2AZ UK fionn.carman18@imperial.ac.uk j.ewen@imperial.ac.uk; b Department of Mechanical Engineering, University of Bath Claverton Down Bath BA2 7AY UK

## Abstract

Accurate prediction of thermophysical properties is crucial for the rational design of lubricants and cooling fluids. We compile a database of approximately 4700 critically evaluated reference values for the density, viscosity, and thermal conductivity of 835 unique organic molecules in the C_10_–C_50_ range. We use this database to train and benchmark a range of machine learning (ML) architectures and molecular representations. XGBoost models trained on molecular descriptors are consistently among the most accurate across all three target properties. Out-of-distribution tests show that descriptor-space distance and the position of the predicted value within the training range identify higher-risk regions at the population level, rather than ordering the errors of individual molecules. Equilibrium molecular dynamics (EMD) simulations of eight representative molecules reproduce density accurately but show systematic, chemistry-dependent force-field errors for the viscosity and thermal conductivity. At an inference cost orders of magnitude lower than EMD simulations, the ML models are well suited to high-throughput screening of new candidate molecules for lubricants and other formulated products.

## Abstract 2

Machine learning models trained on ∼4700 reference values predict the density, viscosity, and thermal conductivity of lubricant-sized organic molecules, benchmarked against Green–Kubo molecular dynamics simulations of eight representative fluids.

## Graphical abstract

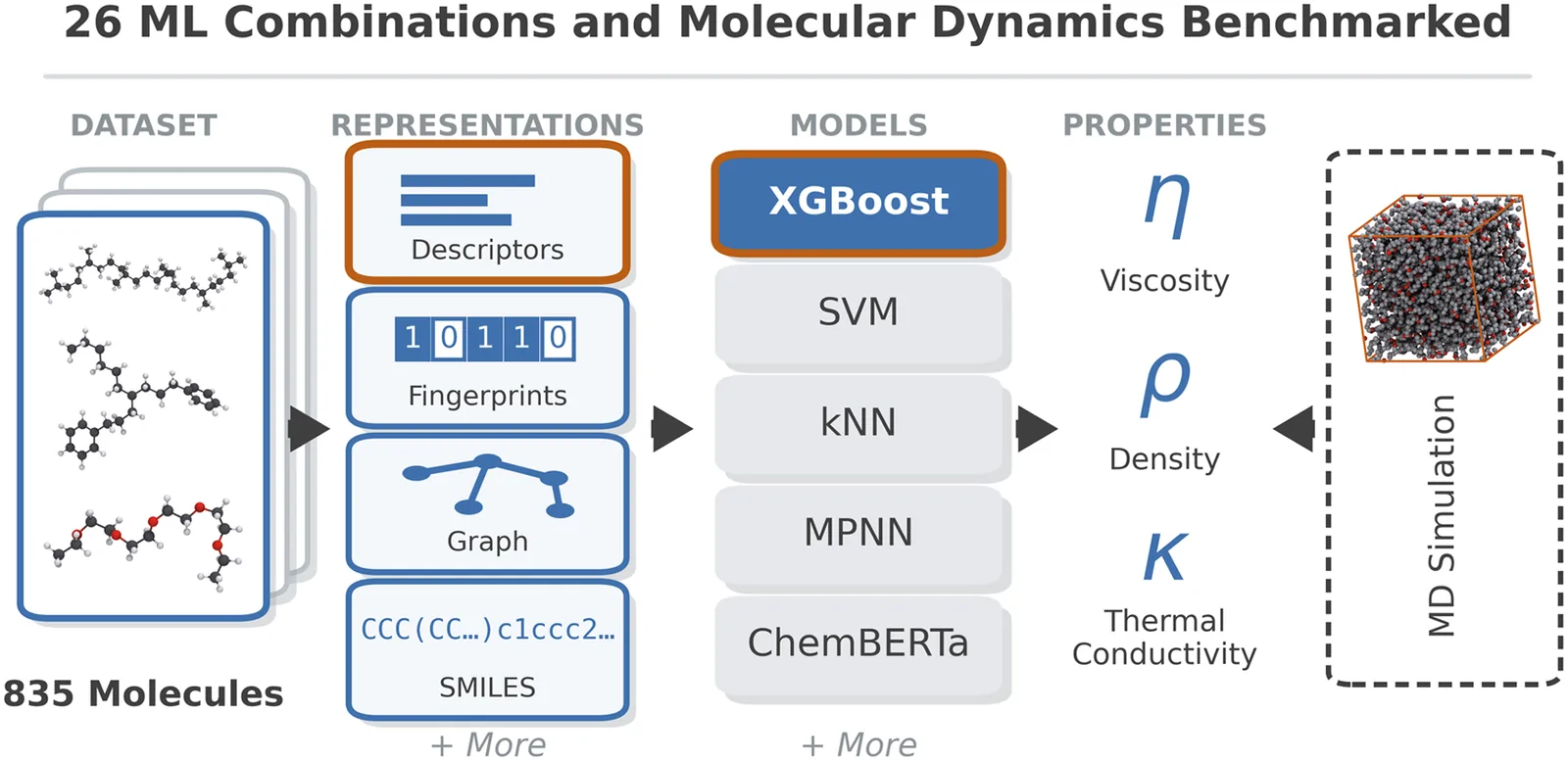

## Introduction

Thermophysical properties such as the density, viscosity, and thermal conductivity of organic molecules are important to the performance of liquid formulations such as lubricants and refrigerants.^1^ Accurate prediction of these properties for pure organic molecules is also important for a wide range of industries, including energy, chemicals, and pharmaceuticals.^2–4^ In this context, the lack of reliable thermophysical data is one of the largest stumbling blocks in rational molecular,^5^ formulation,^6^ and process^7^ design. In particular, lubricants are required to adhere to strict viscosity limits across a wide temperature range (40–100 °C);^8^ however, for the organic molecules relevant to this application (C_10_–C_50_),^9^ viscosity–temperature data are still lacking. Viscosity data are available for many normal alkanes up to ∼C_24_, but data for branched alkanes, cycloalkanes, aromatics, alkyl cycloalkanes, and alkyl aromatics is much more sparse.^10^ There is also a lack of reliable thermophysical data available for oxygen-containing molecules such as esters, ethers, and glycols in the C_10_–C_50_ range.^11^ It is often possible to interpolate between missing entries with calculated predictions using a reliable equation of state, such as the perturbed-chain statistical associating fluid theory (PC-SAFT).^12,13^ Several other predictive models are available for pure hydrocarbons,^14^ with the most promising being group contribution and particularly entropy scaling methods.^15,16^ Quantitative structure–property relationship (QSPR) models^17–21^ have also been identified that are capable of predicting the viscosity of organic liquids. Within the chemical families and conditions for which they are parameterized, these methods can be highly accurate; their main limitation is reduced transferability, with predictive performance degrading outside the molecules and conditions for which experimental data are available.^14^ This makes extrapolation beyond the experimentally gathered dataset to new molecules or conditions less reliable. A quantitative comparison between these physically based methods and the machine learning models benchmarked here would require method-specific parameterization across the full dataset, and is left for future work.

Over the last few decades, molecular dynamics (MD) simulations have become established as a useful tool with which to predict thermophysical properties and generate reliable datasets.^22^ MD simulations can in principle be applied to any organic liquid for which the molecular structure is known and a suitable force field is available, with an accuracy set by the force field and the sampling.^23,24^ The accuracy of force fields for MD simulations of the thermophysical properties of small molecules^25^ and linear and branched hydrocarbons has recently been studied.^26^ However, this type of comparison is still lacking for other industrially important classes of organic molecules, such as those containing cycloalkane, aromatic, ester, and ether groups. MD simulations are now routinely used to calculate the shear viscosity of single-component,^26–29^ binary mixtures,^30–34^ and multi-component^35–37^ hydrocarbon fluids with high accuracy over a wide range of conditions. In most cases, viscosity calculations through MD simulations have been restricted to small molecules, typically linear or branched alkanes. This is because larger hydrocarbons generally have higher viscosities,^38^ which are more challenging to accurately quantify through MD simulations due to their longer relaxation times.^39^ While MD simulations have also been used to predict the density and viscosity of oxygen-containing molecules^40,41^ and their mixtures,^42^ there are far fewer studies than for aliphatic hydrocarbons.

Machine learning (ML) has also emerged as a useful tool for predicting the thermophysical properties of pure organic liquids^43^ and their mixtures.^44^ Accurate viscosity prediction has been achieved using ML models such as artificial neural networks for single-component,^45–51^ binary mixtures,^52^ and multi-component^53,54^ organic liquids. ML methods have also been shown to be effective in predicting the viscosity of more complex molecules, such as ionic liquids^55^ and deep eutectic solvents.^56^ As with QSPR models, the performance of ML models remains at the mercy of accurate training data availability, with small, sparse or noisy datasets all having adverse effects on model accuracy. Even the largest available datasets on the thermophysical properties of fluids, such as the NIST REFPROP database,^57^ are sparse and subject to considerable uncertainties compared to other popular applications of ML.^43^

Some recent studies have combined MD and ML by using dynamic descriptors from the MD simulations to improve ML predictions of various fluid properties.^58,59^ In other cases, MD simulations have been used to generate reliable datasets with which to train ML models that can then be used for fluid property prediction.^60,61^ MD simulations have also been used to generate large databases of viscosity values, which have then been used to optimize the molecular structure of lubricants with high viscosity index using ML methods.^5^ A combination of MD simulations and ML has also been used to facilitate the data-driven design of polymer-based viscosity index improver additives.^62^ A recent study compiled a large experimental database containing viscosity values for 6120 small organic liquids^59,63^ and used this to train ML models to predict viscosity.^64^ They found that the ML prediction models outperformed MD simulations across a range of molecules and temperatures.^64^ A combination of ML and MD simulations was recently employed to predict the density, kinematic viscosity, thermal conductivity, specific heat capacity, and dielectric constant of esters in the context of immersion cooling fluids.^65^ Both MD simulations^66^ and ML models^67^ have been considered for thermophysical property prediction for decades; however, only relatively recent advances in computational hardware and software have made this type of wide parameter study feasible.^22^ However, systematic side-by-side evaluations of the two approaches on common sets of fuel- and lubricant-sized organic molecules remain scarce.

In this study, we benchmark ML models for the prediction of the density, viscosity, and thermal conductivity of lubricant-relevant organic liquids, and use MD simulations on a representative subset as an independent, physics-based assessment of the same properties. The primary aim is to develop ML models suitable for high-throughput virtual screening^62,68^ and molecular discovery^5,69^ workflows for high-performance lubricants. The MD simulations serve a complementary purpose, probing where and why physics-based prediction succeeds or fails for these types of molecules.

We evaluate 26 combinations of model architecture and molecular representation on approximately 4700 critically evaluated reference values for 835 fuel and lubricant-sized molecules, to identify the most accurate combination for each property. Because screening is most valuable beyond the chemistries that have already been measured, we then examine how far that accuracy extends to molecules unlike those seen in training, and which signals available at prediction time indicate where it does not. Finally, we apply Green–Kubo MD simulations to eight molecules, two from each of the four principal chemical families of the dataset, at 40 °C and 100 °C. This extends force field benchmarks that have concentrated on linear and branched alkanes^26,28,29^ to alkyl cycloalkanes, alkyl aromatics, esters, and ethers, for which experimental viscosity data remain sparse^10^ despite their importance in automotive fuels,^70^ aviation fuels,^71^ and lubricants.^9,72^

## Methodology

### Machine learning models

To construct a robust and diverse dataset for model development and evaluation, we collected reference data for density, thermal conductivity and dynamic viscosity at atmospheric pressure and at both 40 °C and 100 °C. Only molecules with a dynamic viscosity of approximately 1–50 mPa s at 40 °C were included, because this is the range accessible to equilibrium molecular dynamics (EMD) simulations.^39^ Data were compiled from the Landolt–Börnstein series^73^ and the NIST Chemistry WebBook^74^ (accessed June 2024), resulting in 835 unique molecules carrying approximately 4700 critically evaluated reference values after the structure canonicalization, unit conversion and deduplication steps described below.

In cases where the properties of interest were not provided exactly at 40 °C or 100 °C, interpolation was used to obtain the values at these temperatures. The NIST WebBook^74^ utilizes a combination of property estimation methods to interpolate liquid viscosity values from the available experimental data. These include the PPDS9 (Physical Property Data Service) and ViscosityL correlations, which are based on semi-empirical and theoretical equations of state (extended Andrade models^75,76^) fitted from experimental values.^4^ The source method of every label is recorded, and the number of labels obtained by direct measurement *versus* by each database's own correlation or reference–property model is reported by property and temperature in Table S2. Most of the labels for density and thermal conductivity (99.0% and 97.8% at 40 °C) and approximately 44% of the viscosity labels are values produced by the source databases' own correlation, interpolation or reference–property models rather than direct measurements. The errors reported in this work therefore measure agreement with these adopted reference values, and do not include the uncertainty of the underlying database correlations. For molecules where both a direct measurement and a correlation value are available, the two agree closely across the full viscosity range at both temperatures (Fig. S1).

For interpolation of the liquid density values, the NIST WebBook uses the Tait equation.^77^ For liquid thermal conductivity, the TransportPolynomial model was applied, utilizing temperature-dependent polynomial fits to experimental thermal conductivity data. For the Landolt–Börnstein series,^73^ cubic spline interpolation using a minimum of three surrounding data points was employed to estimate the property values at the desired temperatures. In cases where only the kinematic viscosity was reported, it was converted to dynamic viscosity using *µ* = *ν* × *ρ*, where *µ* is dynamic viscosity, *ν* is kinematic viscosity, and *ρ* is density. Molecules whose viscosity was reported only as kinematic and for which no density was available at the same temperature were excluded (283 molecules at each temperature). Canonicalization and deduplication of the 1187 source entries give 1114 unique molecules, of which 835 retain at least one usable label and form the dataset used here. The viscosity values were normalized by taking their natural logarithm to improve regression performance during ML training.^54^

A summary of the reference dataset is presented in Table 1. Of the 835 unique molecules, 637 (76%) are pure hydrocarbons containing only carbon and hydrogen, while the remaining 198 (24%) are oxygen-containing organic compounds such as esters and ethers. Oxygen-containing molecules were included alongside the pure hydrocarbons because oxygenated lubricant-like molecules, such as synthetic esters,^78^ are an important part of the target application space, and both classes are represented in the MD benchmark below. The chemical family composition of the reference dataset is summarized in Table S1, with each molecule assigned to a single chemical family, as recorded in the released dataset. Some molecules may belong to more than one family due to the presence of multiple functional groups.

**Table 1:** Summary of the critically evaluated reference dataset compiled from the Landolt–Börnstein series^73^ and NIST Chemistry WebBook.^74^ The dataset contains thermophysical properties for 835 unique organic molecules

Property	No. of compounds	Min. value^a^	Max. value^a^
Density (40 °C)	770	0.715	1.400
Density (100 °C)	769	0.668	1.340
Dynamic viscosity (40 °C)	799	0.54	56.30
Dynamic viscosity (100 °C)	804	0.29	22.00
Thermal conductivity (40 °C)	785	0.054	0.276
Thermal conductivity (100 °C)	786	0.051	0.250

a Units for density, viscosity and thermal conductivity are g cm^−3^, mPa s and W m^−1^ K^−1^, respectively.

In this study, we compare the most commonly used molecular representations on our reference dataset to see which enables ML models to perform best on transport property prediction tasks. Fig. 1 shows the different molecular representations used to train ML models in this study.

**Fig. 1:**
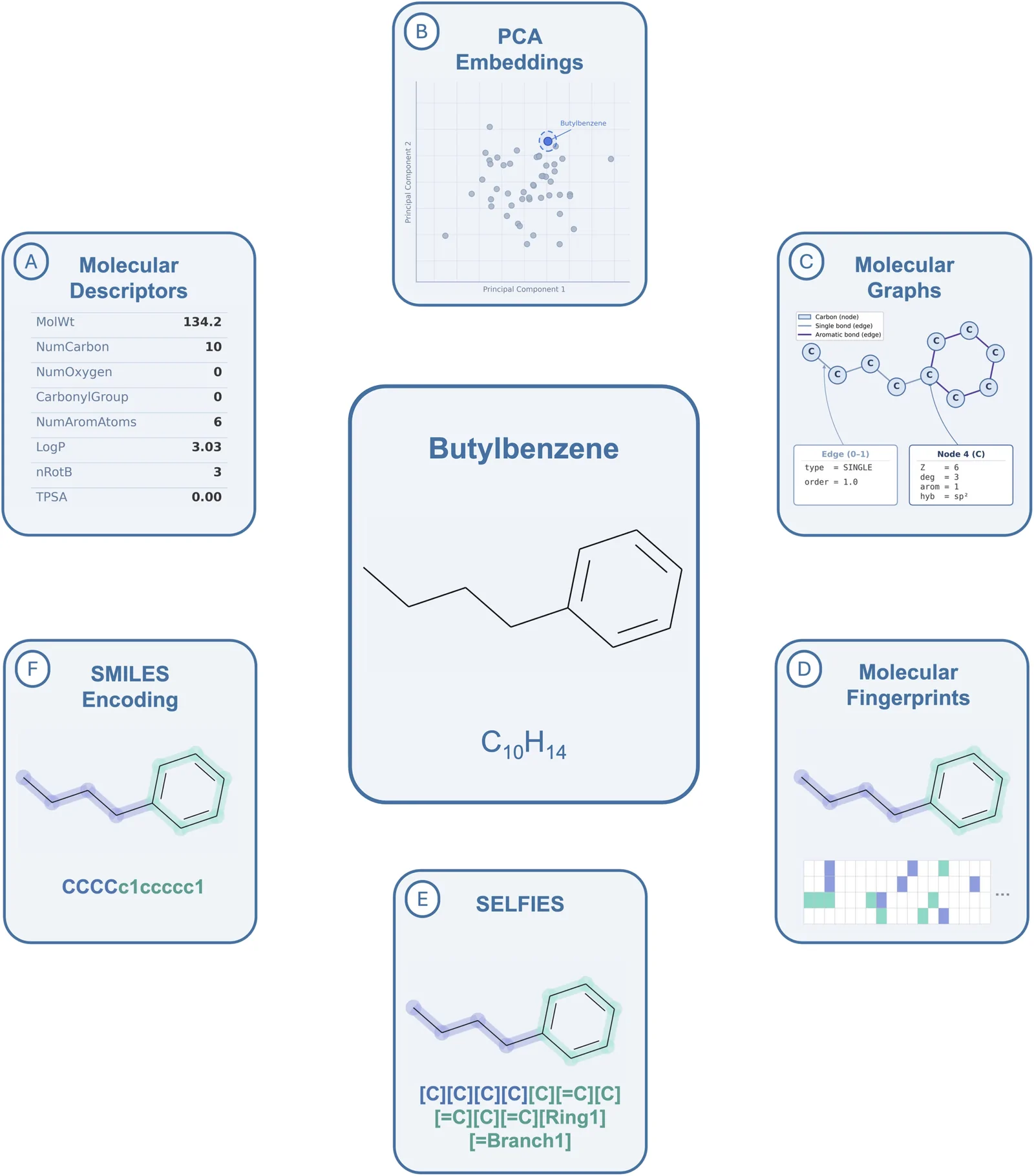
The molecular representations used to train the ML models, illustrated for butylbenzene 138%.

Molecular descriptors are numerical features that encode chemical, physical, and structural properties of molecules and are widely used for ML-based property prediction.^79,80^ In this study, descriptors were generated using the RDKit and Mordred Python libraries,^80^ yielding 1019 descriptors per molecule after removal of invalid, missing, and categorical features. Descriptor scaling was applied selectively based on model requirements. *Z*-score normalization was used for magnitude-sensitive models (SVM and *k*-NN), while no scaling was applied for tree-based models (random forest and XGBoost) to preserve the natural feature distributions and chemically meaningful thresholds.

Dimensionality-reduced embeddings provide a compact alternative to high-dimensional molecular descriptors by reducing noise and redundancy while retaining the most informative features.^81^ In this study, embeddings generated using principal component analysis (PCA) and uniform manifold approximation and projection (UMAP) were evaluated across a range of dimensionalities to assess their impact on model performance, as both the choice of method and retained dimensionality can influence predictive accuracy. PCA and UMAP embeddings were used as inputs to ML models to compare linear and nonlinear dimensionality reduction strategies for molecular property prediction. A detailed analysis of embedding method and dimensionality effects, including root-mean-square error (RMSE) trends across properties and model types, is provided in the SI. Embeddings were generated using the scikit-learn and UMAP-learn libraries from *z*-score normalized molecular descriptors.

Molecular fingerprints are typically represented as 2D binary strings, where each bit indicates the presence or absence of a specific structural feature or substructure within a molecule. A widely used and generally high-performing fingerprint type is the extended-connectivity fingerprint (ECFP), with the Morgan fingerprint being a common example.^82^ In this study, ECFPs are generated using the Morgan Fingerprint implementation in RDKit.^79^ While prior work has examined optimal ECFP parameters for molecular similarity assessment,^83^ no studies have specifically focused on optimizing ECFP generation for the property prediction of fuel- and lubricant-sized organic molecules using ML models. Accordingly, this study evaluates a range of ECFP generation parameters to identify those that yield the best regression performance. A systematic analysis of fingerprint parameters, including radius, bit size, and hashed *versus* unhashed representations,^83^ is provided in the SI.

Molecular graphs encode information about molecules by representing atoms as nodes and the bonds between them as edges. Nodes can be characterized by atom-level features such as atomic number, while the edges can incorporate bond-related characteristics such as bond type. Table S3 shows the features used to create molecular graphs in this study.

Simplified molecular-input line-entry system (SMILES) strings encode molecular structures as sequences of ASCII characters^84^ but are not inherently unique, as multiple valid strings can represent the same molecule. Canonical SMILES address this by enforcing a deterministic representation,^85^ although models trained on canonical SMILES may overfit to syntactic patterns rather than underlying chemistry.^86^ Despite this, SMILES remain widely used due to their simplicity and compatibility with sequence-based models such as CNNs and transformers.^87^ SELFIES (Self-Referencing Embedded Strings) were introduced to guarantee syntactic validity, ensuring that any token sequence corresponds to a chemically valid molecule.^88^ In this study, both canonical SMILES and SELFIES were evaluated as input representations for molecular property prediction. Strings were converted to token embeddings prior to model training; SMILES were generated from IUPAC names,^89^ and SELFIES were generated using the published SELFIES API.^88^

Pretrained transformer embeddings were obtained from ChemBERTa,^90,91^ a chemistry language model pretrained on large SMILES corpora. The frozen ChemBERTa-77M-MTR encoder was used as a fixed feature extractor. Each molecule's SMILES string is passed through the pretrained encoder and the token representations are mean-pooled (weighted by the attention mask) into a single fixed-length vector, with no fine-tuning of the encoder weights. These frozen embeddings were used as inputs to a multilayer perceptron (MLP) head and to an XGBoost model, providing a transfer-learning representation that complements the descriptor-, fingerprint-, and string-based inputs above.

In this study, we evaluate a range of models using the collated dataset to identify the most suitable algorithm for property prediction. The classic ML models, including support vector machines (SVMs),^92^ random forests (RFs),^93^ and *k*-nearest neighbors (*k*-NN),^94^ are implemented using the scikit-learn package in Python.^95^ XGBoost (Extreme Gradient Boosting) models were trained using the XGBoost Python library.^96^ The graph-based models, message passing neural networks (MPNNs)^97^ and graph attention networks (GATs),^98^ are implemented with the PyTorch Geometric library.^99^ The deep learning models, convolutional neural networks (CNNs),^100^ multilayer perceptrons,^101^ and transformers^87^ were implemented with the PyTorch library.^99^ ChemBERTa^90,91^ was evaluated in two configurations using the HuggingFace Transformers library: fine-tuning the full pretrained encoder end-to-end on SMILES, and the frozen-embedding configuration described above, in which only a small MLP head is trained on the fixed mean-pooled encoder output.

All models were evaluated under a single shared nested cross-validation protocol, applied to molecular representation configurations that were fixed in advance. The embedding dimensionality and the fingerprint radius and bit size were chosen by grid search on a single 80 : 10 : 10 train/validation/test split, as reported in the SI, so the protocol is nested with respect to hyperparameter selection and conditional on those representation configurations. The dataset was partitioned into five fixed outer folds, so that both temperatures of a given molecule always fall in the same fold and no molecule appears in more than one outer test set. The folds were constructed by stratified sampling over quintiles of the log-transformed 40 °C viscosity labels (molecules without a viscosity label were assigned *via* median-filled strata), then frozen. For each outer fold, hyperparameters were selected by random search over 25 configurations sampled from model-specific ranges, scored on a designated validation partition of the non-test data (approximately 85 : 15 train/validation); the selected configuration was then retrained on all non-test data and evaluated once on the untouched outer test fold. Full details of the architectures, hyperparameter ranges, and training procedures for each model are provided in the SI.

### Molecular dynamics simulations

The molecular structures of the eight molecules selected for this benchmarking study are shown in Fig. 2. These molecules were chosen to span the principal chemical families encountered in the reference dataset used in this work. All the MD simulations were performed using the LAMMPS software.^102^ EMD simulations were employed to calculate the density, viscosity, and thermal conductivity of the eight target molecules at 40 °C and 100 °C, consistent with conditions used to determine lubricant viscosity index values.^8^ All simulations were performed using the LOPLS-AA force field.^103^ Parameters for the LOPLS simulations were taken from Jorgensen *et al.*^104^ and Siu *et al.*^103^ Where required, the ester extension to LOPLS-AA was employed,^105^ while aromatic and ether parameters were taken from the corresponding OPLS-AA parameterizations. LOPLS-AA was selected because previous benchmarks have demonstrated it to be among the most accurate all-atom force fields for reproducing the experimental density and viscosity of linear hydrocarbons, including *n*-hexadecane,^28^*n*-dodecane, and *n*-octacosane.^29^

**Fig. 2:**
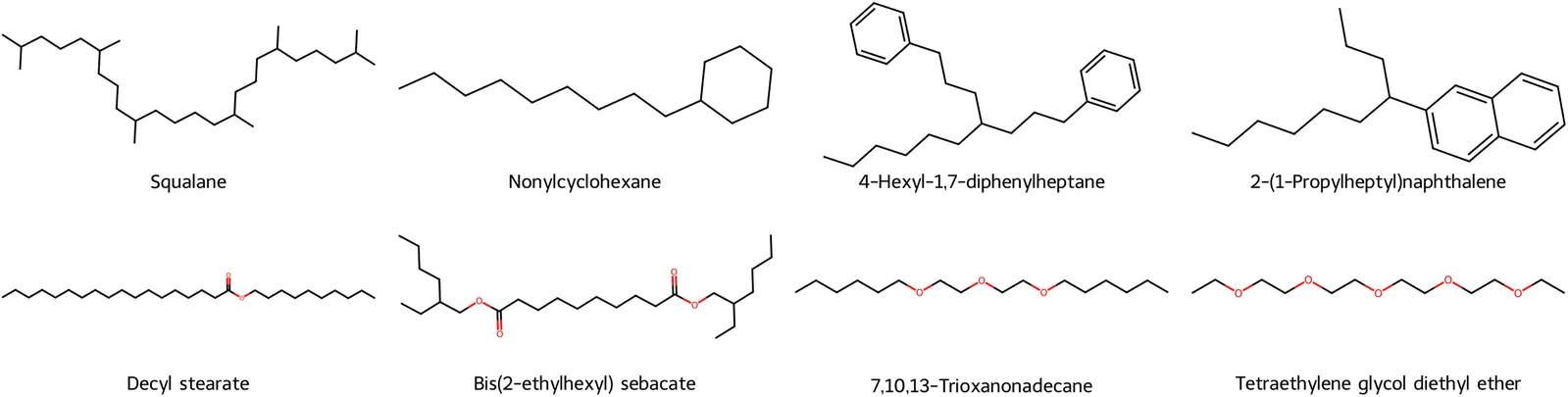
The eight MD target molecules, two from each principal chemical family of lubricant base oils in the dataset: alkanes (squalane, nonylcyclohexane), alkyl aromatics (4-hexyl-1,7-diphenylheptane, 2-(1-propylheptyl)naphthalene), esters (decyl stearate, bis(2-ethylhexyl) sebacate), and polyethers (7,10,13-trioxanonadecane, tetraethylene glycol diethyl ether).

Initial single molecule geometries were generated from the SMILES strings in RDKit by adding explicit hydrogens, embedding with the ETKDGv3 distance geometry algorithm,^106^ and minimizing with UFF. Cubic starting configurations were assembled with Packmol^107^ at a low initial density (0.3 g cm^−3^), with molecule counts selected to give approximately 15 000 atoms per box (163–349 molecules, depending on molecular size), corresponding to equilibrated cubic box lengths of 5.2–5.5 nm. A dedicated system size sensitivity study was not performed here, since all systems are well above the range where finite-size effects would be expected to affect the viscosity^39^ and thermal conductivity.^108^ Periodic boundary conditions were applied in all three Cartesian directions.

The protocol suggested by Schmitt *et al.*^26^ was adopted in the EMD simulations because it has been shown to enable accurate calculation of the viscosity of lubricant-sized molecules. A velocity–Verlet integrator with a 1.0 fs timestep was employed.^109^ Bonds containing hydrogen atoms were constrained with the SHAKE algorithm.^110^ Constraining C–H bonds is important for obtaining accurate thermal conductivities with all-atom force fields, as it suppresses the classically over-excited high-frequency C–H stretching modes that otherwise inflate the Green–Kubo heat flux.^111^ Lennard-Jones interactions were truncated at 12 Å with long-range tail corrections applied to energy and pressure, and long-range electrostatics were treated using the particle–particle particle–mesh (PPPM) method^112^ with a relative accuracy of 10^−5^. Geometric mixing rules were used for the unlike-atom interactions, and intramolecular 1–4 interactions were scaled by 0.5 following the OPLS convention.

The system was first relaxed by conjugate gradient energy minimization, followed by a short displacement-capped Langevin dynamics stage to remove any remaining close contacts. An equilibration in the canonical (*NVT*) ensemble was then performed at the target temperature to allow the system to reach thermal equilibrium. The density was then equilibrated at 1.0 atm and the target temperature for 1.0 ns in the isothermal–isobaric (*NPT*) ensemble using the Nosé–Hoover thermostat and barostat^113,114^ with damping coefficients of 100 fs and 1000 fs, respectively. The density was obtained directly as the ensemble averaged mass density at atmospheric pressure over the final 0.25 ns of the *NPT* trajectory, where a steady-state value had been reached.^26^ Density uncertainties were estimated as five-block standard deviations over this averaging window and are at most 0.003 g cm^−3^ (≤0.4%) for every state point. The simulation box was then deformed isotropically over 0.5 ns at constant temperature until the fluid density matched this average value. For each molecule, 10 independent replicas were branched from the equilibrated configuration with independently seeded velocities, each given a 0.2 ns *NVT* warm-up before production. Production was carried out in the *NVT* ensemble for 8 ns per replica to allow the slow shear-stress autocorrelation tail to converge.^26^

Viscosity was calculated using the Green–Kubo (GK) formalism,^115,116^ which is well suited to low and moderate viscosity fluids (<50 mPa s).^39^ The GK expression for the shear viscosity is:1
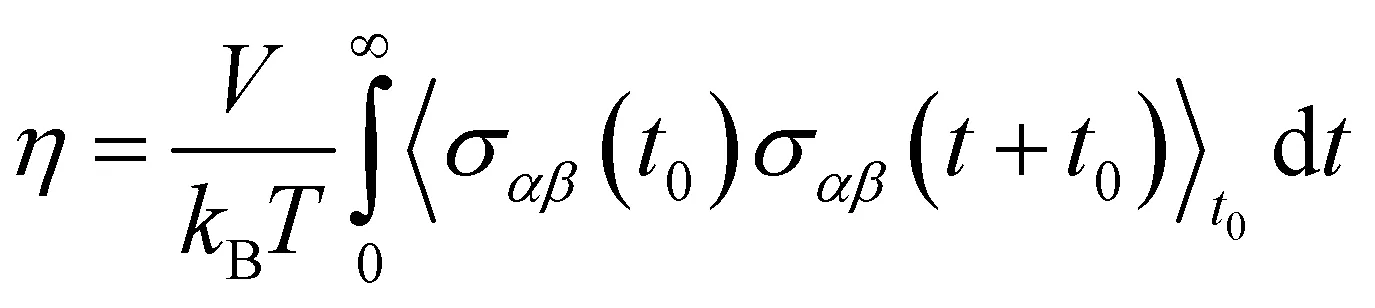
where *η* is the viscosity, *V* is the system volume, *k*_B_ is the Boltzmann constant, *T* is the temperature, and *σ*_*αβ*_(*t*) is an off-diagonal component of the stress tensor at time *t* (with *α* and *β* denoting Cartesian directions *x*, *y*, or *z*); the angle brackets denote the stress autocorrelation function (ACF) averaged over time origins. To maximize the statistical sampling, the viscosity was averaged over all six independent components of the pressure tensor, combining the three off-diagonal terms with the three diagonal-difference terms, as recommended by Maginn *et al.*^39^

The time decomposition method (TDM) of Zhang *et al.*^117^ was used to improve the reliability of the calculations for the high-viscosity molecules. The time evolution of the running integral *η*(*t*) was recorded over the full production trajectory of each replica, with the stress ACF evaluated using a fast Fourier transform (FFT) technique.^118^ A cutoff time *t*_cut_ was applied to the integral to mitigate accumulation of long-time noise, determined from the ratio of the standard deviation *σ*_*η*_(*t*) across replicas to the average running viscosity:^117^
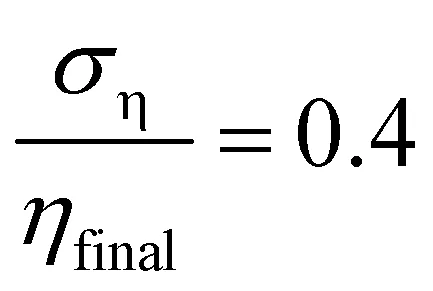
matching the criterion of Schmitt *et al.*,^26^ who showed that ratios of *σ*_*η*_/*η*_final_ between 0.2 and 0.8 do not materially affect the extracted values. The bootstrapping method,^119^ with 200 iterations, was used to compute the final viscosity value and its uncertainty, following the time-decomposition procedure of the PyLaT software^120^ and implemented in the released analysis script. Random combinations of the 10 replicas were resampled with replacement, and for each combination the replica-averaged running viscosity *η̄*(*t*) was fitted with the double-exponential function of eqn (2), in which *η*_∞_ is the long-time viscosity limit and *α*, *β*_1_, and *β*_2_ are fitting parameters:2
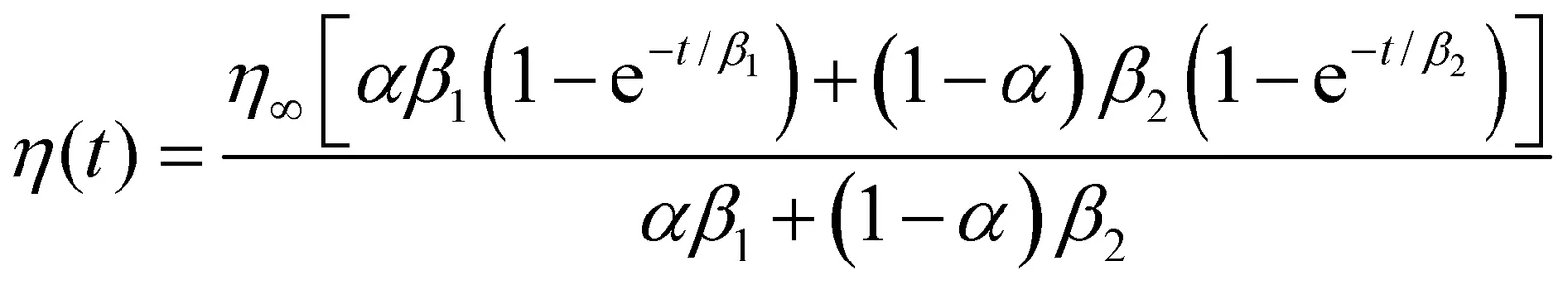


This functional form reflects the physics of the underlying stress autocorrelation function, which decays through two distinct regimes: a fast relaxation associated with local, high-frequency stress fluctuations and a slow relaxation governed by collective, long-time structural rearrangement.^117^ The two exponential terms, with time constants *β*_1_ and *β*_2_ and relative weight *α*, capture these two modes, so that the running integral *η*(*t*) rises from zero and saturates toward the plateau value *η*_∞_, which is taken as the converged viscosity. Fitting to this form allows the well-sampled short-time behavior to determine *η*_∞_ while avoiding the accumulation of statistical noise in the poorly converged long-time tail of the integral, thereby providing a more robust estimate than reading off the running integral directly.^39,117^

The Green–Kubo formalism was also used to compute the thermal conductivity *κ*,^121,122^ from the autocorrelation of the microscopic heat flux vector **J**_q_:3
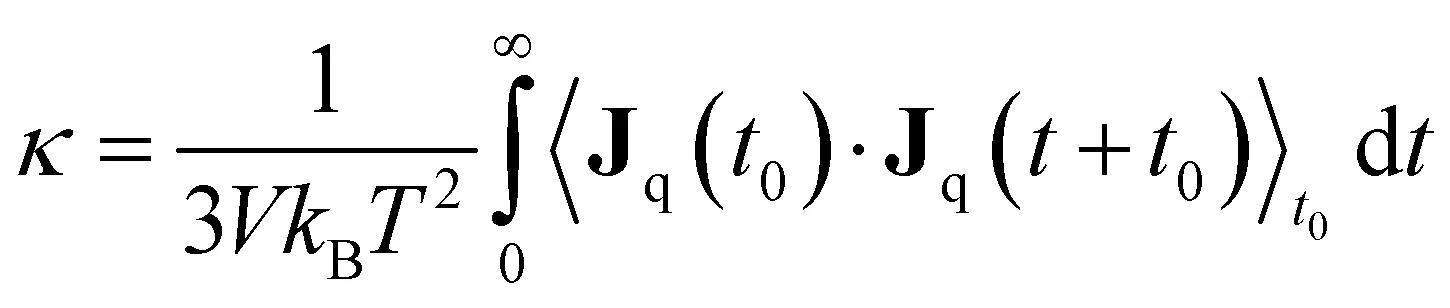
where *κ* is the thermal conductivity, **J**_q_ is the heat flux vector, the factor of three averages over the Cartesian components of the heat flux, and the angle brackets indicate a time-origin average of the autocorrelation function. The microscopic heat current was evaluated in LAMMPS using the centroid-based per-atom stress formulation of Surblys *et al.*^123^ for the pair, bond, angle, dihedral, and improper virial contributions. This formulation treats the many-body bonded interactions consistently in the heat-current expression, which is essential for obtaining accurate thermal conductivities in molecular systems with angle, dihedral, and improper terms.

As with the viscosity, the thermal conductivity was obtained from the running Green–Kubo integral *κ*(*t*), with the heat-flux ACF averaged over the three Cartesian components and evaluated by FFT.^118^ Unlike the shear stress, however, the heat-flux ACF carries no slow tail. The ACF decays to zero within a few picoseconds, so the running integral *κ*(*t*) reaches an early plateau and then drifts as statistical noise accumulates in the long-time tail. The double-exponential TDM fit used for the viscosity is therefore not required for the thermal conductivity, and integrating *κ*(*t*) to long lag times would only accumulate noise; the uncertainty of the Green–Kubo estimate grows with the upper integration limit relative to the total trajectory length,^39^ so the conductivity must be read from the short-time plateau rather than the converged integral. To locate this plateau without a hand-chosen cutoff, we first identified the lag time t* at which the normalized heat-flux ACF decays into its noise floor, defined as the first point beyond which its magnitude remains below twice the standard deviation of the long-time, noise-dominated tail. The thermal conductivity was then taken as the average of *κ*(*t*) over the window [t*, t* + 10 ps], immediately after the autocorrelation has decayed and before long-time noise sets in, with the reported value and uncertainty given by the mean and standard deviation of this window average across the 10 independent replicas.

## Results and discussion

### Comparing ML models

Prior to benchmarking, we investigated embedding and fingerprinting strategies by systematic grid search on a single 80 : 10 : 10 train/validation/test split, with full details provided in the SI (Fig. S2–S5, Tables S4 and S5). For embeddings, PCA consistently outperformed UMAP across most properties and model types; one hypothesis is that its linear variance-preserving transformations suit the axis-aligned splits used by tree-based models, though the benchmark does not test this directly. Based on these results, PCA embeddings of 100 dimensions were used for all models. For fingerprints, increasing radius and bit size improved performance despite higher bit-collision rates, and unhashed Morgan fingerprints with a radius of 8 were selected as they eliminated collision-induced ambiguity and yielded the best overall predictive performance. Both comparisons rest on that single split and were not repeated within each outer fold, so they rank embedding and fingerprint configurations under one split rather than under the nested protocol.

All 26 model–representation combinations were then evaluated under the shared nested cross-validation protocol described in the Methods, using the same fixed outer folds for every model, representation and property. Conditional on the representation configurations fixed above, the quoted test errors are therefore free of test-set-tuned hyperparameter selection, although the outer folds serve as a comparative evaluation across the 26 combinations rather than a fully untouched holdout for the single winning model. All metrics reported below are means over the five outer folds, with the between-fold standard deviation as an estimate of their variability.

Table S6 reports the mean test RMSE and *R*^2^ for every model–representation combination, property and temperature; per-fold metrics, including mean absolute error (MAE) and the between-fold standard deviations, are provided with the released code. To identify the strongest models we *z*-score normalize the per-property RMSE values, invert them so that lower error corresponds to higher performance, and average across properties; we use this ranking only to select a common set of leading models and base all substantive claims on the property-specific results.

Fig. 3 shows how the five best-ranked models perform for each property at 40 °C (a–c) and 100 °C (d–f). Here “PCA embeddings” refers to the 100-dimensional PCA of molecular descriptors described above, whereas “ChemBERTa embeddings” refers to the fixed mean-pooled vectors produced by the pretrained ChemBERTa encoder.^90,91^

**Fig. 3:**
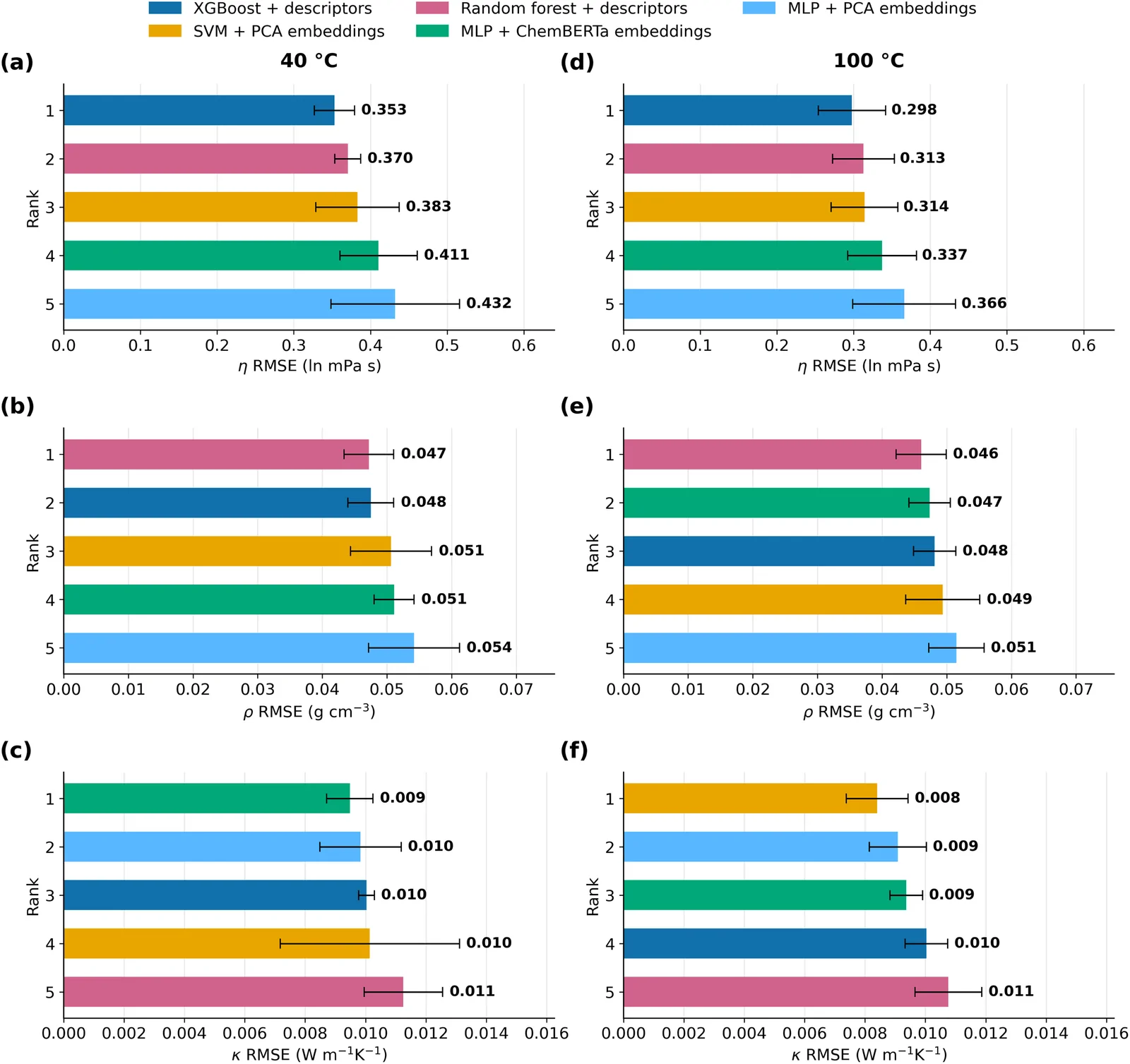
The five best-ranked ML model–representation combinations for each property at 40 °C (a–c) and 100 °C (d–f): (a and d) viscosity, modeled as ln(*η*/mPa s), (b and e) density and (c and f) thermal conductivity. Bars are mean test RMSE over the five locked outer folds, ordered by rank, with between-fold standard deviations as error bars. “PCA embeddings” denotes the 100-dimensional PCA of molecular descriptors and “ChemBERTa embeddings” the frozen mean-pooled ChemBERTa vectors. Results for all 26 combinations are given in Table S6.

XGBoost and RF models, when used with molecular descriptors, performed most consistently across all three transport properties. XGBoost with descriptors gave the lowest viscosity error of any combination (RMSE = 0.35 and 0.30 in ln(*η*/mPa s) at 40 and 100 °C, *R*^2^ = 0.86 and 0.83) and was among the best two or three combinations for density and thermal conductivity, while RF with descriptors gave the lowest density error (RMSE = 0.047 g cm^−3^). An error of 0.35 on the logarithmic scale corresponds to a typical discrepancy of a factor of 1.4 between predicted and measured viscosity. This aligns with previous studies,^124,125^ which also found tree-based models to be reliable across datasets. These methods handle nonlinear relationships and high-dimensional features effectively, making them suitable for the diverse information captured by molecular descriptors, and are relatively insensitive to feature scale, irregular target functions and uninformative features.^126^

When comparing representations for XGBoost, overall predictive performance followed the trend descriptors > PCA embeddings > ChemBERTa embeddings > fingerprints, and this ordering is particularly pronounced for viscosity, where the fingerprint model was substantially weaker (RMSE = 0.67 and 0.55; *R*^2^ = 0.51 and 0.42). This may be explained by the fact that while descriptors and PCA embeddings can incorporate both structural and physicochemical information, fingerprints are purely structural, lacking explicit encoding of global molecular properties, which limits their expressiveness for the transport properties considered here.

The SVM and *k*-NN models with molecular descriptors also achieved accuracy close to the leading tree-based models. SVM with descriptors reached a viscosity RMSE of 0.38 and 0.32 (*R*^2^ = 0.84 and 0.80), while *k*-NN with descriptors was clearly weaker at 0.44 and 0.36. For these distance-based models, descriptor and PCA embedding inputs gave comparable accuracy (for example, SVM viscosity RMSE of 0.38/0.32 with descriptors *versus* 0.38/0.31 with PCA embeddings). Some sensitivity to the high dimensionality and redundancy of descriptor sets is expected for distance-based methods,^127^ and may explain their larger errors relative to the tree-based models.

The GAT and MPNN models, trained on molecular graphs augmented with three-dimensional geometric information (bond lengths and partial charges derived from ETKDG conformers), were the weakest combinations, with GAT giving a viscosity RMSE of 0.78 and 0.63 (*R*^2^ = 0.33 and 0.22). This is notable given the theoretical advantages of geometric information for modeling molecular properties, and that graph ML methods have shown strong performance on related tasks.^52,128^ Two factors plausibly contribute: graph models typically require larger datasets than the one used here,^129^ and ETKDG conformers,^106^ while inexpensive, do not always capture the most energetically favorable geometry of flexible molecules, so the bond lengths and partial charges may only approximate the environments that govern transport properties. More accurate conformer generation, using density functional theory or machine-learning force fields, is therefore one route to improvement. Within this study the MPNN outperformed the GAT for every property and temperature, by 19 and 26% in viscosity RMSE at 40 and 100 °C and by 4 to 15% for density and thermal conductivity, on identical graphs and under the same search budget, although both remained well behind the descriptor-based models.

Among the deep-learning models, the transformer trained on the PCA molecular embeddings fell in the lower third of the benchmark (*η* RMSE 0.53 and 0.39; *R*^2^ = 0.69 and 0.70), well behind the MLP trained on the same embeddings (0.43 and 0.37), so the shortfall lies with the architecture rather than the representation. The two ChemBERTa^91^ configurations behaved very differently. Used as a frozen feature extractor, with only a small MLP head trained on the fixed mean-pooled embedding of each molecule, ChemBERTa was one of the strongest models overall. It gave the lowest thermal-conductivity error at 40 °C (RMSE = 0.0095 W m^−1^ K^−1^, *R*^2^ = 0.89) and consistent performance across all three properties, with viscosity accuracy (RMSE = 0.41 and 0.34) moderately behind the leading descriptor models. By contrast, fine-tuning the full ChemBERTa encoder end-to-end on SMILES predicted viscosity somewhat more accurately (RMSE = 0.37 and 0.31) but was markedly less consistent, with substantially larger density and thermal-conductivity errors (*ρ R*^2^ = 0.51). This pattern suggests that, on a dataset of this size, updating all encoder weights can overwrite useful pretrained features, whereas freezing them and learning only a lightweight head preserves the transferable representation and generalizes better.

A chemistry-pretrained transformer was thus among the best models for these transport properties when its pretrained embeddings were used directly. The comparatively poor SMILES fine-tuning is consistent with a distributional mismatch between ChemBERTa's predominantly drug-like pretraining corpus^91,130^ and the lubricant-sized molecules studied here, and points to parameter-efficient fine-tuning such as Low-Rank Adaptation (LoRA),^131^ which freezes the base weights and introduces small trainable matrices, as a promising alternative, as demonstrated for other large chemistry and protein language models.^132^

Models trained on character-level embeddings of string-based molecular representations such as SMILES and SELFIES generally underperformed relative to models using molecular descriptors or PCA embeddings, though the best string-based models (the CNN and transformer) were only moderately behind the leading combinations for viscosity. For a given architecture, SMILES inputs matched SELFIES for viscosity (for example, CNN RMSE of 0.45/0.35 for SMILES *versus* 0.45/0.36 for SELFIES) and tended to outperform them for density and thermal conductivity, despite the theoretical advantages of SELFIES in guaranteeing valid molecular strings. This performance gap may stem from differences in how the two formats encode structure. While SMILES strings are not guaranteed to produce valid molecules when randomly generated or mutated,^85^ they tend to preserve local structural patterns such as rings, branches and functional groups, so that substructures that are adjacent in chemical space often remain close in the string; this implicit locality may help character-level models learn chemically meaningful sub-patterns. SELFIES strings, by contrast, are designed always to decode to valid molecules,^88^ but the encoding can disrupt local chemical continuity, place related substructures far apart in the sequence, and produce longer strings with larger token vocabularies, all of which can make it harder for convolutional or recurrent models to learn useful patterns from token proximity. Throughout, these comparisons characterize the particular implementations, feature choices and tuning budget evaluated here under a common protocol, rather than the model classes in general; the graph and sequence models were the most sensitive to those choices in our hands.

The property-specific *R*^2^ values in Table S6 reveal several further trends across the leading models. Density has the lowest *R*^2^ of the three properties for every model (*R*^2^ ≤ 0.77), but this reflects the narrow spread of the density labels rather than poor accuracy: the best model reaches 0.047 g cm^−3^, about 5% of the mean value and the smallest relative error of the three properties. For thermal conductivity and viscosity, moderate-to-high *R*^2^ values are observed, with the descriptor-based tree models and, for thermal conductivity, the frozen ChemBERTa embeddings performing best. These trends highlight how both temperature and feature representation affect regression accuracy across the different thermophysical properties.

The viscosity results are also robust to the source method of the training labels. Retraining the XGBoost descriptor models using only the explicitly experimental viscosity labels, with the same locked folds and selected hyperparameters, left the error on directly measured test labels unchanged at 40 °C (RMSE 0.277 *versus* 0.272) and marginally lower at 100 °C (0.209 *versus* 0.225), well within the fold-to-fold variability (Table S7). The recovered correlation and interpolation labels, approximately 44% of the viscosity training data, therefore neither inflate nor degrade accuracy on measured data.

For thermal conductivity and density an equivalent check is not possible, because all recovered labels for these properties derive from the source databases' correlation models; the reported errors therefore measure agreement with those critically evaluated reference values. The associated temperature interpolation is, however, far better conditioned than for viscosity. The median change between 40 °C and 100 °C across the dataset is 4.4% for density and 5.9% for thermal conductivity, compared with a median factor of 3.5 for viscosity.

### Out-of-distribution prediction

It is well-known that ML models yield unreliable property predictions for molecules that lie outside the chemical domain of their training sets.^133,134^ This limitation poses significant challenges for molecular discovery pipelines,^135^ where candidate compounds frequently occupy regions of chemical space not sampled during training, and experimental measurements for such molecules are scarce. The severity of the problem depends on representation. Fingerprint-based models encode only substructures seen during training, so novel fragments activate no informative bits, while string-based tokenizations rely on sequential relationships that out-of-distribution atoms and functional groups distort. Molecular descriptors vary smoothly with structural modification and encode continuous global quantities that can change more gradually than sparse substructure indicators, making them comparatively better suited to extrapolation.^136^ Tossou *et al.* characterized out-of-distribution (OOD) prediction as a covariate shift problem and showed that performance degradation depends more on representation than on choice of model, with RDKit descriptors minimizing it.^137^

Quantifying OOD status is itself difficult. Previous studies have used Tanimoto similarity on fingerprints^138^ or Euclidean distance in descriptor space,^137^ but novel compounds trigger near-zero fingerprint similarity, and Euclidean distance can be dominated by a few high-variance axes. More fundamentally, any distance measures where a molecule sits relative to the training inputs; it cannot detect when the property value itself lies beyond the training labels. We therefore evaluate four signals, each available at prediction time, for the XGBoost descriptor viscosity model: Mahalanobis distance, which normalizes descriptor scales and captures inter-feature correlations,^139^ mean 5-nearest-neighbor distance in the same descriptor space, mean Tanimoto distance on Morgan fingerprints, and cross-fold disagreement, the standard deviation across models trained on different subsets of the training data.

The two descriptor-space distances are computed for the 100 descriptors ranked most important by each fold's trained model ensemble. To probe behavior beyond the training distribution we assembled an OOD test set of 40 lubricants with measured viscosities, none present in training: 21 containing atom types absent from training (N, P, S, Si) and 19 with viscosities above the training range (Tables S8 and Table S9). Signals are referenced to the training distribution as percentiles where noted, and all in-domain evaluations use out-of-fold (OOF) predictions.

At the population level every signal tracks error (Fig. 4). Binned by signal percentile, OOF RMSE rises toward the highest percentile bins for every signal and at both temperatures, with the size of the increase varying by signal and temperature; the largest is for Mahalanobis distance at 40 °C, where the RMSE grows from 0.20 in ln(*η*/mPa s) in the lowest decile to 0.59 in the 95–100th percentile bin. At the level of individual molecules the same signals carry little information. Spearman correlations between signal and error are 0.27 to 0.42 (Table S10). The signals therefore act as population-level indicators of where errors are likely to be larger, rather than error estimates for individual molecules.

**Fig. 4:**
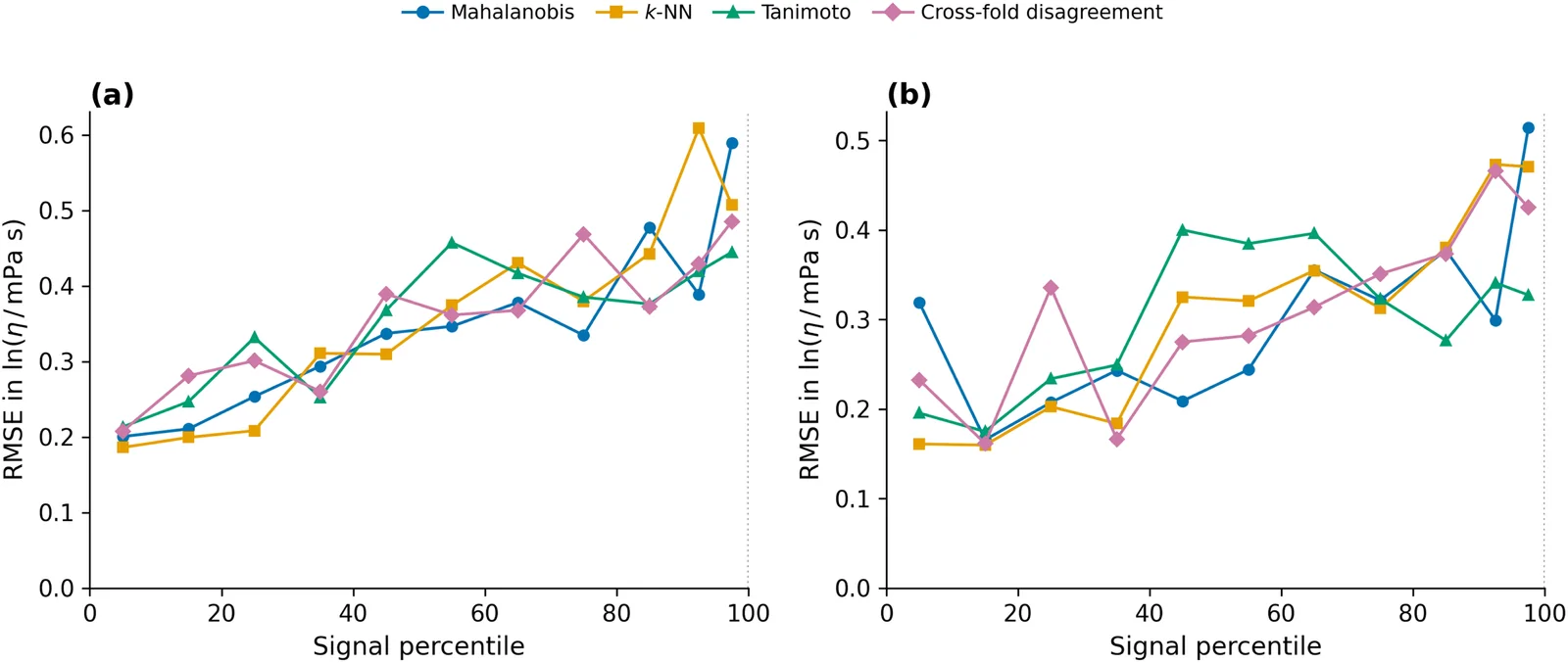
OOF RMSE against signal percentile for the four applicability-domain signals at (a) 40 °C and (b) 100 °C. Bins are deciles with the top decile split at the 95th percentile.

Fig. 5 plots the absolute percentage error of each OOD molecule against each raw signal, overlaid on the corresponding OOF errors of the training molecules. Most OOD molecules sit above the training cloud, but within the OOD set no signal ranks error (|*r*_s_| ≤ 0.15 on percentage errors). Distance and error are not correlated. The most chemically distant heteroatom molecules are often predicted well, while the largest errors belong to high-viscosity molecules that look ordinary to every signal.

**Fig. 5:**
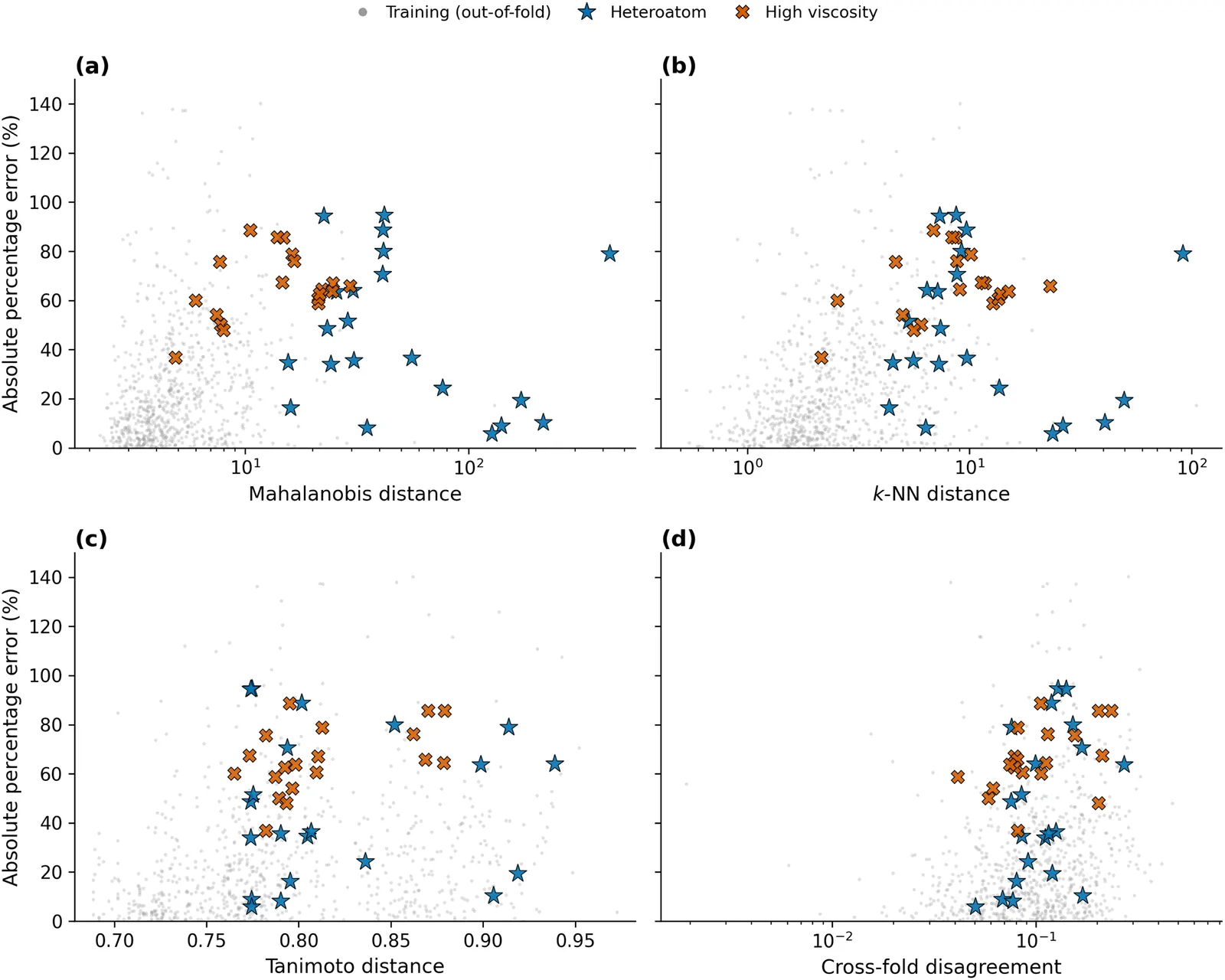
Per-molecule absolute percentage error in viscosity against each raw signal at 40 °C: (a) Mahalanobis distance, (b) *k*-NN distance, (c) mean Tanimoto distance, (d) cross-fold disagreement. Gray points are OOF predictions for training molecules, and the two categories of the OOD set are those containing an element absent from the training molecules (heteroatom) and those whose measured viscosity exceeds the training range (high viscosity). The Spearman rank correlation between signal and percentage error, taken over the training and OOD molecules together, is 0.36 for Mahalanobis distance, 0.39 for *k*-NN distance, 0.30 for Tanimoto distance and 0.26 for cross-fold disagreement.

This behavior follows from a known limitation of tree-based models. Piecewise constant trees do not extrapolate trends beyond their training data, and the fitted XGBoost model saturates near the maximum of its training labels. Since 18 of the 19 high-viscosity molecules have measured *η*_40_ above the training-label maximum of 56.3 mPa s, their predictions fall short, by 34 to 88% here, regardless of how familiar their chemistry is. The heteroatom molecules carry no such systematic shortfall. Their chemistry is new, but their viscosities lie inside the training range, so the model can still land near the right value, and its errors vary from molecule to molecule. The parity plots in Fig. S6 show the effect directly. At 40 °C the high-viscosity molecules sit almost entirely beyond the training range on the experimental axis, and their predictions form a horizontal band between 20 and 35 mPa s instead of following the unity line. At 100 °C all but one of them fall back inside that range and return to the diagonal, and their RMSE improves from 1.25 in ln-units to 0.60 (Table S11, Fig. S7 and S8).

The two problems are caught by different coordinates (Fig. 6). New chemistry shows up as distance, but only in descriptor space. Mahalanobis distance flags all 21 heteroatom molecules and *k*-NN distance flags 17 of them, while Tanimoto distance and cross-fold disagreement flag only 5 and 1 (Table S12), and on the Mahalanobis panel the distance flag alone catches 34 of the 40 OOD molecules. The range limitation is instead identified from the model's own output, since predictions near the training maximum are the ones at risk of being capped. A flag at the 90th percentile of the training labels catches the remainder, so the two flags together catch all 40 while flagging 15% of the training set (Table S12). Both are available at prediction time.

**Fig. 6:**
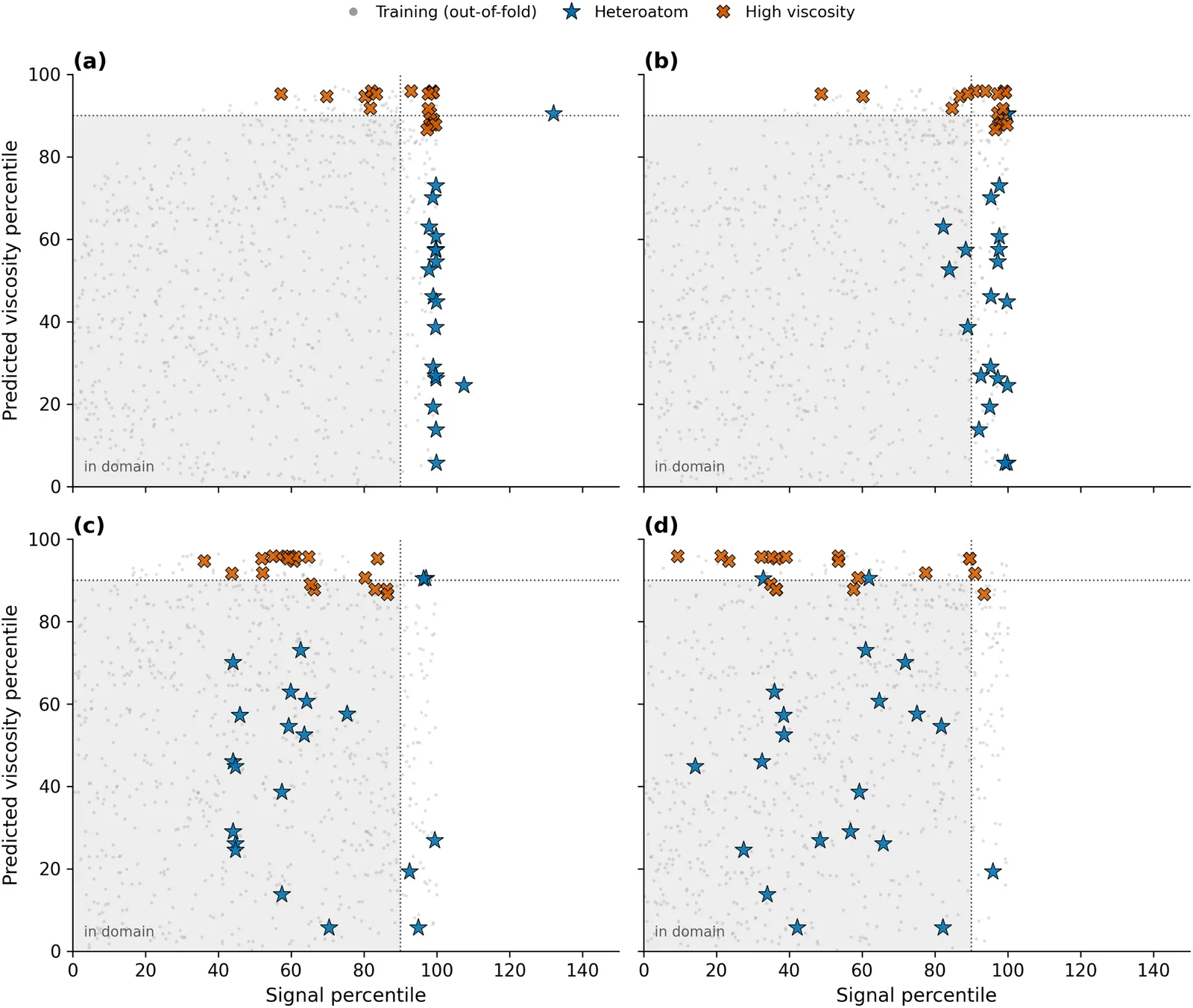
Predicted viscosity, as a percentile of the training labels, against signal percentile at 40 °C for (a) Mahalanobis distance, (b) *k*-NN distance, (c) Tanimoto distance, and (d) cross-fold disagreement. Signal percentiles above 100 are extended in proportion to the largest training value, and the axis is limited to 150. Dotted lines mark the 90th percentiles and the shaded rectangle is the joint in-domain region. The numbers of heteroatom and high-viscosity molecules falling outside it are 21 of 21 and 19 of 19 in (a), 17 of 21 and 19 of 19 in (b), 5 of 21 and 14 of 19 in (c), and 3 of 21 and 15 of 19 in (d). Two molecules in (a) lie beyond the axis limit and are not shown.

None of these signals alone provides a reliable per-molecule error estimate, and none of them orders the errors of the individual OOD molecules. Taken together they separate groups of molecules with a higher mean error from groups with a lower one. A molecule is flagged as OOD when a distance signal places it beyond the training distribution, or when its predicted value approaches the end of the training range for the property, placing it in a higher-risk group identified by this retrospective analysis rather than establishing that its prediction is individually extrapolative or quantitatively invalid. Combined at the 90th percentile, the Mahalanobis and predicted-value flags catch every molecule of the OOD set, and over the pooled training and OOD molecules the flagged group carries 1.8 to 2.1 times the mean absolute percentage error of the unflagged group (42 *versus* 24% at 40 °C and 37 *versus* 18% at 100 °C; Table S13). Fig. S9 shows that the mean error rises steadily from the center of the domain toward its boundaries, with the flagged OOD region representing the upper end of this gradual trend rather than a distinct change in behavior. These signals therefore identify regions in which the average error is higher, rather than quantifying or ordering the error of an individual prediction. The two-signal rule was formulated and evaluated on the same external set, so it is exploratory and would require independent validation before being used as a predictive reliability criterion.

### Molecular dynamics

We complement the ML models with EMD simulations of the eight target molecules shown in Fig. 2, spanning the four principal chemical families in the reference dataset with two molecules from each. Density, viscosity, and thermal conductivity were computed at 40 and 100 °C with the LOPLS-AA force field,^103^ assigned directly from each molecule's SMILES string using our open-source builder tool. The same properties were predicted with the best-performing XGBoost descriptor models identified in the ML benchmarking above. The comparison is not intended to adjudicate one method against the other, but to characterize MD as an independent, physics-based route to these properties and to weigh the strengths and limitations of each. Per-molecule MD, ML, and reference values for all three properties at both temperatures are tabulated in Tables S14–S16.

Of the three properties, density is reproduced most accurately. MD matches the reference values to within 3% for six of the eight molecules at 40 °C (within 4% for seven) and within 6% for seven of the eight at 100 °C (Fig. 7a and S10a), consistent with previous benchmarks for linear and branched alkanes.^26^ The instantaneous density reaches a stable plateau within approximately 0.2 ns of the 1 ns *NPT* equilibration (shown for all eight molecules at both temperatures in Fig. S11), and no residual equilibration drift is detectable in the reported values.

**Fig. 7:**
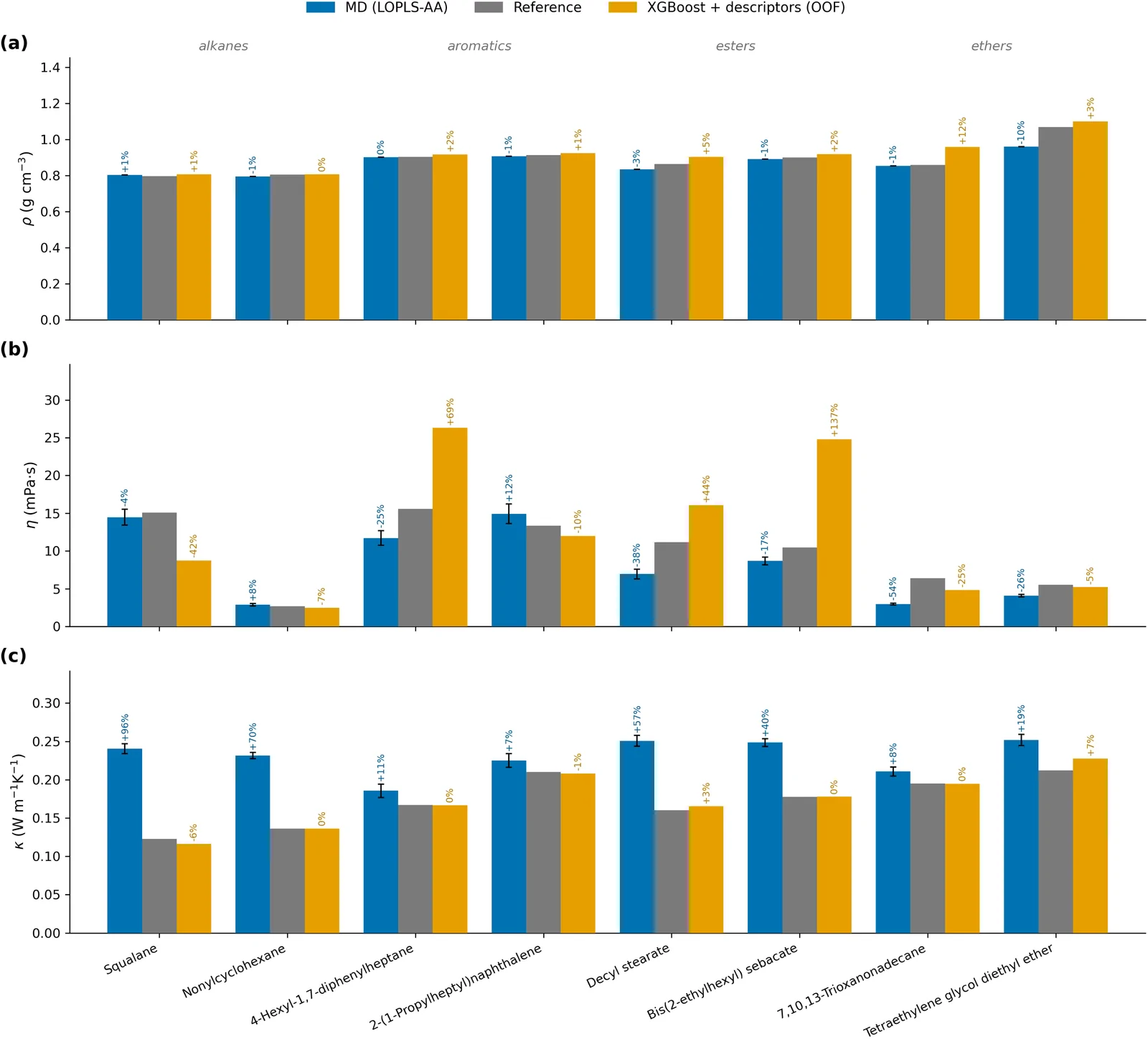
MD (LOPLS-AA, Green–Kubo), reference, and XGBoost predictions for the eight target molecules at 40 °C: (a) density, (b) dynamic viscosity, and (c) thermal conductivity, grouped by chemical family. Reference values are obtained by correlation or interpolation from the source databases (see Methods). Labels give the percentage deviation of MD and XGBoost from the reference values. MD error bars are standard deviations over the ten replicas (bootstrap for viscosity). The XGBoost values are OOF predictions, for which the test RMSE is a global rather than per-molecule uncertainty, so no error bars are shown.

The exception is tetraethylene glycol diethyl ether, the most oxygen-rich molecule of the set, which MD under-predicts by 10% at 40 °C and 13% at 100 °C. This agreement is expected, since liquid densities are governed largely by the repulsive and dispersion parameters and force fields such as OPLS are often directly fitted to reproduce liquid densities.^103,104^ The XGBoost density predictions carry errors of similar magnitude but opposite character. No molecule is missed badly, yet deviations of +5 to +12% remain for decyl stearate and 7,10,13-trioxanonadecane, molecules whose chemistry is sparsely represented in the training data, even though their density values lie within the training range.

Transport properties, such as the viscosity, are not usually used as target properties during force field parameterization because they require longer simulations to calculate.^22^ This makes them a more challenging target for transferable MD force fields such as OPLS. Fig. S12 shows the stress autocorrelation function (Fig. S12a) and its running Green–Kubo integral *η*(*t*) (Fig. S12b) for decyl stearate at 40 °C. The slow tail of the autocorrelation means the integral rises over several hundred picoseconds, the double-exponential TDM fit^117^ is taken up to the *σ*_*η*_/*η*_∞_ = 0.4 cutoff, and beyond the cutoff the spread across replicas grows rapidly as statistical noise dominates. To ensure proper convergence, we analyzed *η*_∞_ for trajectory lengths between 1 and 8 ns, showing that *η*_∞_ plateaus at approximately 6–8 ns for all eight molecules (Fig. S13). Accordingly, all production viscosity calculations were performed using 8 ns trajectories to ensure convergence with respect to trajectory length.

The accuracy of the force field varies with the chemistry of the molecule (Fig. 7b and S10b). For the four hydrocarbons, the viscosity deviations from the reference values are generally modest. Squalane deviates by −4% at 40 °C and −7% at 100 °C, nonylcyclohexane by +8% and −1%, 2-(1-propylheptyl)naphthalene by +12% and +14%, while 4-hexyl-1,7-diphenylheptane, under-predicted by 25% at 40 °C, recovers to −5% at 100 °C. By contrast, viscosity is under-predicted for all oxygen-containing molecules at both temperatures. The esters by 17–38% (decyl stearate −38% and −32%; bis(2-ethylhexyl) sebacate −17% and −25%) and the ethers by 26–57% (7,10,13-trioxanonadecane −54% and −57%; tetraethylene glycol diethyl ether −26% and −47%). A similar viscosity under-prediction was also noted for diesters in previous MD simulations^42^ with LOPLS.^105^ Because the trajectory-length sweeps plateau well below the reference values, rather than continuing to rise toward them (Fig. S13), these systematic under-predictions are unlikely to arise from incomplete Green–Kubo convergence. Instead, they are consistent with a limitation of the force field for the oxygen-containing molecules in this dataset. With only eight molecules used for the MD benchmarking, these observations can only be used to identify plausible explanations, rather than establish firm conclusions. Despite these limitations, the force field largely preserves the ordering of the reference viscosity values for the eight fluids at both temperatures, suggesting that MD simulations remain useful for relative screening, even where its absolute viscosity predictions require caution.

The XGBoost viscosity predictions show no comparable chemistry-dependent bias: their deviations scatter in both directions within each family. Their magnitudes are, however, larger than for density or thermal conductivity, and in line with the benchmark's OOF viscosity RMSE of 0.35 in ln(*η*/mPa s), which corresponds to a one-standard-deviation factor of 1.4. Four of the eight molecules lie within 25% of the reference values at 40 °C, with squalane under-predicted by 42% and decyl stearate, 4-hexyl-1,7-diphenylheptane, and bis(2-ethylhexyl) sebacate over-predicted by 44, 69, and 137%; at 100 °C four lie within 10%, with decyl stearate (+45%), 7,10,13-trioxanonadecane (−27%), tetraethylene glycol diethyl ether (−37%), and bis(2-ethylhexyl) sebacate (+144%) the exceptions.

The thermal conductivity converges far more quickly in the MD simulations compared to the viscosity, but it carries the opposite bias. As shown in Fig. S14a, the autocorrelation of the heat current decays within a few picoseconds, so its running integral *κ*(*t*) (Fig. S14b) reaches an early plateau from which the conductivity is read before noise accumulates at longer times; unlike the viscosity, the estimates are already converged within the first 1–2 ns of trajectory (Fig. S15). LOPLS over-predicts *κ* for every molecule at both temperatures, with deviations ranging from +7 to +96% (Fig. 7c and S10c).

The magnitude of the over-prediction increases with the amount of aliphatic C–H bonds across the eight molecules. The saturated alkanes are worst (squalane +96% at 40 °C and +87% at 100 °C; nonylcyclohexane +70% and +74%), the esters follow (decyl stearate +57% and +54%; bis(2-ethylhexyl) sebacate +40% and +50%), the polyethers are milder (+5 to +22%), and the two aromatics, whose hydrogen is carried largely on rigid ring carbons, are mildest (+7 to +11%); across the set the deviations correlate with the number of aliphatic hydrogens per heavy atom (40 °C deviations, *n* = 8: Pearson *r* = 0.79, two-sided *p* = 0.020; Spearman *r*_s_ = 0.81, *p* = 0.015; Fig. S16). The two polyethers are the clearest exceptions, their thermal conductivity is over-predicted by less than their aliphatic hydrogen content alone would imply. This ordering is consistent with a vibrational contribution. Hydrocarbons contain C–H bonds that produce very fast, high-frequency intramolecular stretching and bending vibrations. Standard classical force fields often over-exaggerate the coupling and propagation of energy through these stiff covalent modes without adequate anharmonic damping, leading to a significant overestimation of heat flux.^111^ While this can be improved by constraining the C–H bonds with SHAKE,^110^ (as done in our MD simulations) this removes only the stretching contribution, leaving the bending and torsional modes of the aliphatic hydrogen atoms classical and over-populated.^111^ The introduction of oxygen atoms introduces lone pairs and heavier local masses, shifting the vibrational spectrum. Dipolar oxygen centers increase local vibrational scattering and disrupt long-range phonon-like coherent transfer along the backbone, lowering the simulated thermal conductivity and leaving it closer to the reference values.^140^

In practice, at 40 °C the simulations compress the reference range of 0.12–0.21 W m^−1^ K^−1^ into a band of 0.19–0.25 W m^−1^ K^−1^ and misorder the molecules, placing squalane, which the reference values place as the least conductive of the eight, among the most conductive. Unlike the viscosity, the LOPLS force field does not reproduce even the relative ordering of the thermal conductivities of the eight fluids. The OOF XGBoost values for this set stay within 8% of the reference values at both temperatures and preserve the ordering.

Fig. 8 shows the mean absolute deviation from the reference values for the eight molecules. The two approaches separate cleanly only for thermal conductivity, where the simulations deviate by 39% and every ML model stays below 3%. For viscosity at 40 °C the force field and the XGBoost descriptor model are each closer for four of the eight molecules, and density is predicted to within a few percent by both.

**Fig. 8:**
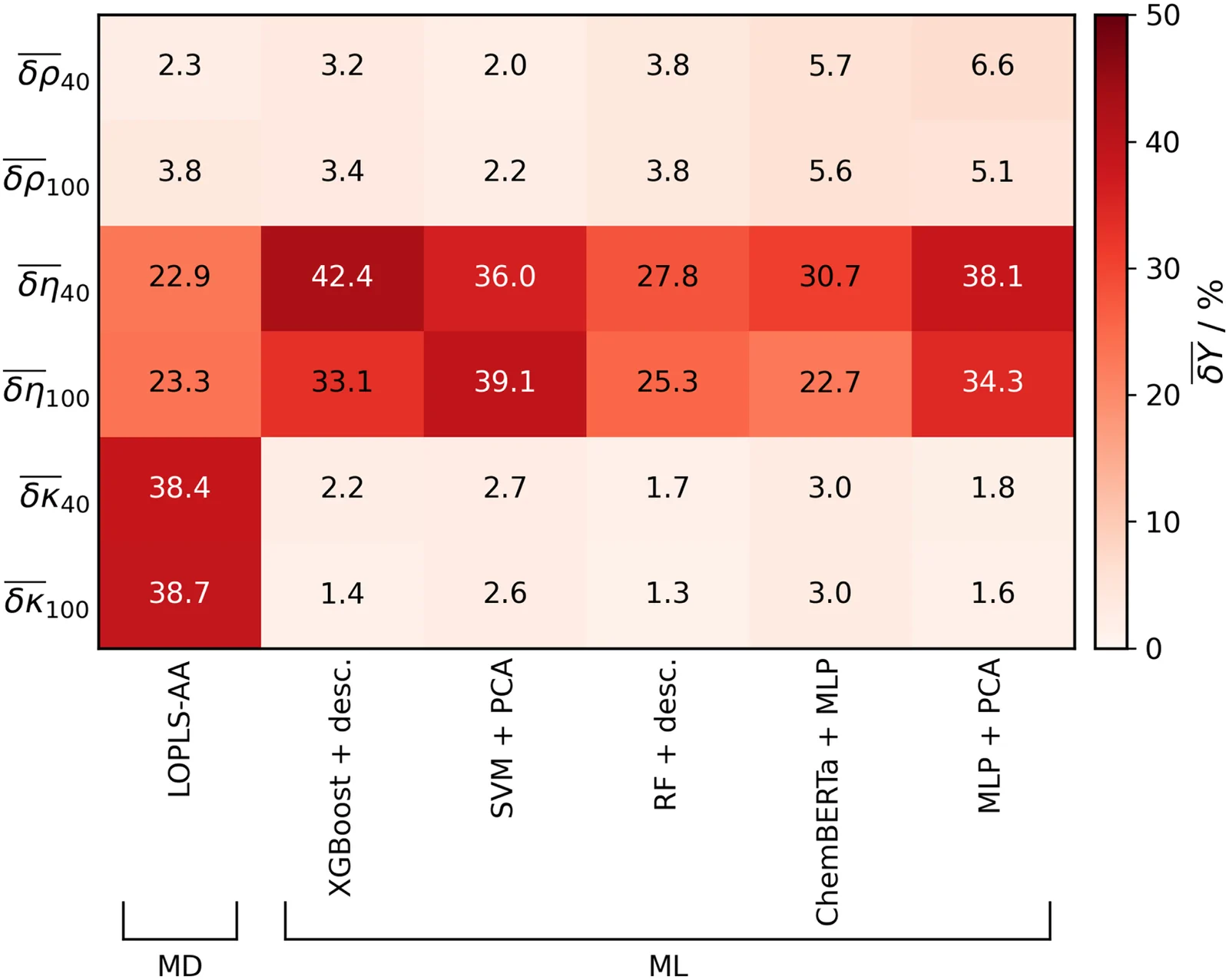
Mean absolute deviation from the reference values for density *ρ*, dynamic viscosity *η* and thermal conductivity *κ*, averaged over the eight target molecules at 40 and 100 °C, for LOPLS-AA and the five best-ranked ML model–representation combinations. Reference values are obtained by correlation or interpolation from the source databases (see Methods).

The two approaches also differ greatly in cost. Converging the transport properties of one molecule at one state point required ten replicas of up to 8 ns each; at the 2.4–3.6 ns per day our systems of ≈15 000 atoms achieve on 24 cores, this corresponds to roughly 1.5 × 10^4^ CPU hours per molecule per temperature, excluding equilibration, or some 2.4 × 10^5^ CPU hours in total for the sixteen state points reported here. Descriptor generation and XGBoost inference for the same molecule take well under a second on a single core. This difference of some seven orders of magnitude is what makes screening libraries of 10^4^–10^6^ candidates feasible for the ML models and out of reach for EMD.

The eight sit toward the edge of the ML training domain, so the descriptor models are tested more stringently here than in the interpolative regime typical of high-throughput screening, where closer agreement with experiment is expected. The benchmark is nonetheless representative of the dataset's chemical families, and both the direction and magnitude of the force-field errors agree with published benchmarks for related fluids.^26,111^ The case study shows that the two methods are suited to different problems. Classical force fields are parameterized primarily for equilibrium thermodynamics, above all liquid density, which they reproduce here to within 4% for seven of eight molecules. On the other hand, these force fields are not fit to transport properties. Their accuracy for viscosity and thermal conductivity is consequently uneven and chemistry-dependent. Viscosity is under-predicted for every oxygen-containing molecule, and thermal conductivity over-predicted proportionally with the aliphatic hydrogen content. Constructing a single force field that is simultaneously accurate for many properties across a broad range of functional groups is therefore very difficult. Conversely, data-driven models can be trained directly on whichever target property has experimental data, at negligible inference cost, which suits it to high-throughput screening where such data exist.

None of this diminishes the role of MD where experimental data cannot reach, such as the non-ideal behavior of mixtures,^30–33,35–37^ or the viscosities at the high pressures that govern friction and film thickness in the elastohydrodynamic lubrication regime,^141^ for which experimental datasets remain far too small (<100 molecules)^142^ to train reliable ML models. We note, however, that standard EMD is itself restricted to fluids of low and moderate viscosity,^39^ so extending predictions to more viscous or glassy regimes requires non-equilibrium methods and remains an open challenge.^141^ In practice, the two approaches occupy different niches: data-driven models for high-throughput screening across the chemistries and conditions where experimental training data exist, and physical simulation for extrapolation beyond them.

## Conclusions

In this study, we built and benchmarked ML models for the prediction of the density, viscosity, and thermal conductivity of fuel- and lubricant-sized organic molecules, and used MD simulations as an independent, physics-based assessment on a representative subset. We evaluated 26 ML model–representation combinations under a shared nested cross-validation protocol on a new critically evaluated reference dataset of 835 molecules, and applied Green–Kubo MD simulations to eight target molecules, two from each of the principal chemical families in the dataset, at 40 and 100 °C.

Our evaluation of molecular representations highlights the importance of representation–model compatibility. PCA-based embeddings outperformed UMAP across most properties and model types, and unhashed Morgan fingerprints with large radii eliminated collision-induced ambiguity and gave the best fingerprint performance; nevertheless, full molecular descriptors remained the strongest representation overall. These two representation comparisons were made on a single split rather than under the nested protocol, and are conditional on it. XGBoost with descriptors gave the lowest viscosity error of any combination and was also among the best models for density and thermal conductivity, with random forests on descriptors best for density and frozen ChemBERTa embeddings with an MLP head best for thermal conductivity at 40 °C. Distance-based models with descriptors followed closely, and a chemistry-pretrained transformer was among the strongest models when its frozen embeddings were used directly.

The MD benchmark, using a Green–Kubo protocol with quantified trajectory-length convergence, shows that the accuracy of the LOPLS-AA force field is property- and chemistry-dependent. Density is reproduced to within 4% for seven of the eight molecules at 40 °C. Viscosity errors are small for the pure hydrocarbons with LOPLS, but this property is consistently under-predicted for the oxygen-containing molecules; the reference ranking of the fluids is nonetheless largely preserved. Thermal conductivity is over-predicted for every molecule, by amounts that correlate with the density of aliphatic C–H bonds, compressing the reference range and misordering the fluids.

The MD case study therefore documents clear differences in force-field accuracy between chemical families, although the small number of molecules means the causes of these differences remain to be firmly established. The ML models, trained directly on the target properties, show no comparable bias across the chemical families of this eight-molecule case study: their OOF predictions stay close to the reference values for the density and thermal conductivity of these molecules, with larger but unsystematic errors for viscosity, at an inference cost some seven orders of magnitude below that of an EMD calculation. The two approaches are therefore suited to different problems: data-driven models for high-throughput screening across the chemistries and conditions where experimental training data exist, and physical simulation for extrapolation beyond them, such as mixtures and elevated pressures, where MD remains indispensable.

We also investigated the OOD prediction capabilities of the ML models, finding that extrapolation beyond the target property range seen during training degrades performance more than encountering molecules with novel atom types. This highlights the importance of training set coverage in both chemical and property space for reliable ML-based screening. The distance and predicted-value signals identify higher-risk regions at the population level rather than ordering the errors of individual molecules, and the two-signal rule remains exploratory because it was assessed on the same external set used to formulate it. We have demonstrated the ability of ML models to accurately predict the thermophysical properties of lubricant base oil-sized molecules, provided that they are trained on high-quality critically evaluated reference data for around a thousand unique molecules, which supports their use as fast property evaluators within multi-objective molecular design workflows for lubricants and other advanced formulations.

## Conflicts of interest

There are no conflicts to declare.

## Supplementary Material

DD-005-D6DD00260A-s001

## Data Availability

The property values used to train and evaluate the machine learning models in this study were obtained from the NIST Chemistry WebBook^74^ and the Landolt–Börnstein series,^73^ and are redistributed with permission. The canonical dataset of 835 molecules (canonical SMILES, IUPAC name, the property labels at 40 °C and 100 °C, and per-label source provenance for every property), the fixed five molecule-level outer folds used for every benchmark in this work, the OOF predictions of all 26 model–representation combinations, their training and feature-generation code, the out-of-distribution test set together with the applicability-domain signals and per-molecule errors behind that analysis, the SMILES-to-LOPLS-AA system builder, LAMMPS input scripts, and Green–Kubo analysis script for the MD case study, and the conda environment specification are available at https://github.com/fionn-carman/MD-ML-thermophysical. The fitted model weights are archived on Zenodo (https://doi.org/10.5281/zenodo.21824121) owing to their size. Supplementary information: chemical family composition of the reference dataset; molecular graph features; optimization of embedding dimensionality and method; optimization of fingerprint radius and bit size; details of ML model architectures, hyperparameter search ranges, and training procedures; per-molecule MD convergence analyses for viscosity, thermal conductivity, and density; per-molecule MD, ML, and reference values for all three properties at both temperatures; OOD test molecule data, signal discrimination and flag-count tables, and 100 °C OOD figures. See DOI: https://doi.org/10.1039/d6dd00260a.
