# Supplementary material for: Benchmarking machine learning and molecular dynamics methods for the prediction of the thermophysical properties of organic liquids
Source: Digit Discov. 2026 Sep 29;5(11):4816–36. doi: 10.1039/d6dd00260a (PMC13647254; doi:10.1039/d6dd00260a)
Supplement: Supplementary file 2 [file DD-005-D6DD00260A-s001.pdf]

Supplementary Information (SI) for Digital Discovery.
This journal is © The Royal Society of Chemistry

Supporting Information: Benchmarking Machine Learning and Molecular Dynamics Methods for the Prediction of the Thermophysical Properties of Organic Liquids

Egheosa Ogbomo,[†,‡] Fionn Carman,[†,‡] Daniele Dini,[†] and James P. Ewen[*,†,¶]

†*Department of Mechanical Engineering, Imperial College London, South Kensington Campus, London SW7 2AZ, U.K.*

‡*These authors contributed equally*

¶*Department of Mechanical Engineering, University of Bath, Claverton Down, Bath BA2 7AY, U.K.*

E-mail: j.ewen@imperial.ac.uk

Methodology

Dataset Compilation

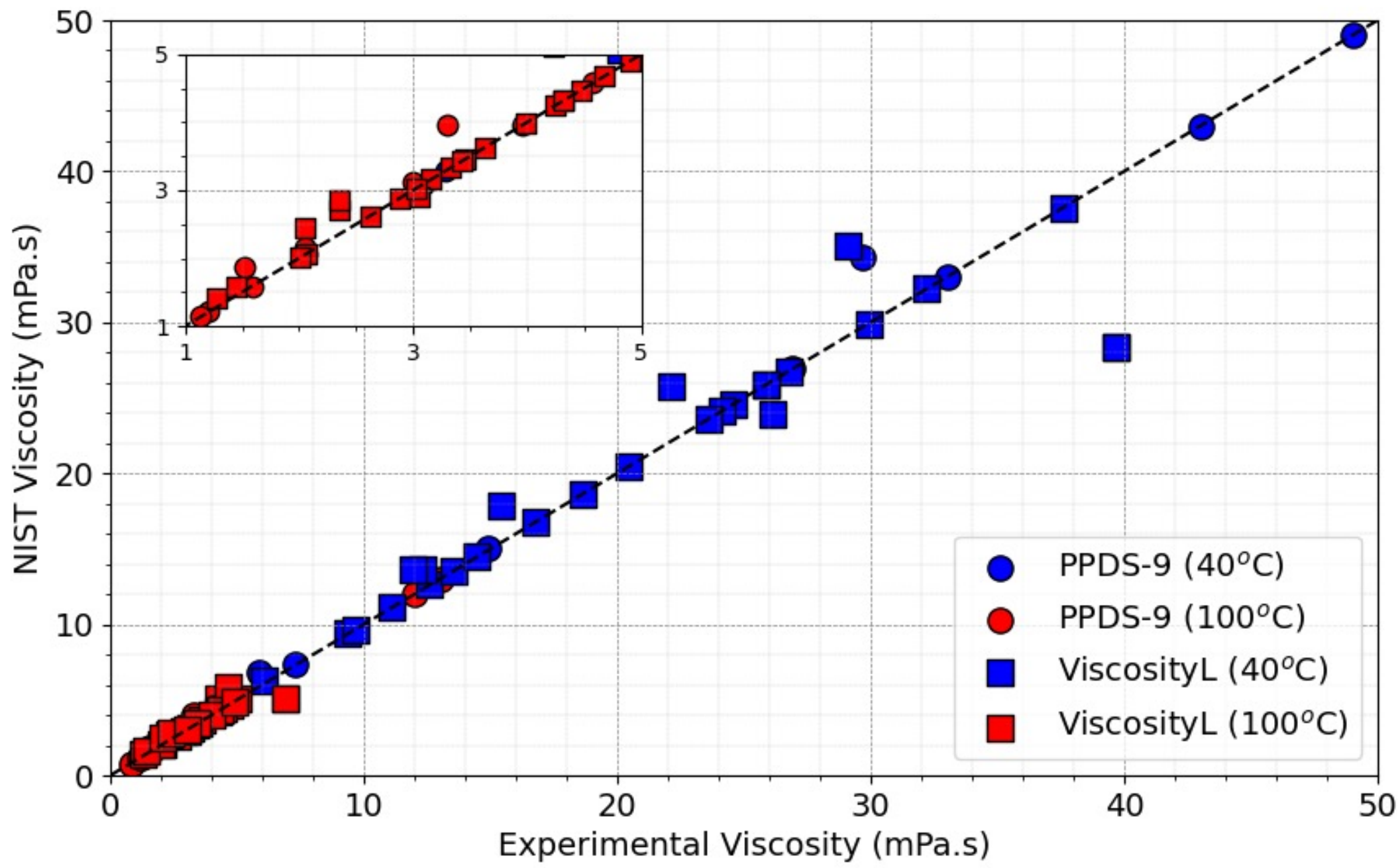

Figure S1: Parity plot of correlation-recovered viscosities from the National Institute of Standards and Technology (NIST) WebBook (PPDS-9 and ViscosityL) against directly measured values at 40 °C and 100 °C, for molecules where both are available. The inset enlarges the region between 0 and 5 mPa s.

Molecular Representations

Molecular Graphs

Support Vector Machines

Support vector machines (SVMs) are widely used ML models suitable for both classification and regression tasks.[2] They are effective in high-dimensional spaces and can handle both linear and nonlinear relationships through the use of kernel functions. The core principle of an SVM is to identify a decision boundary (hyperplane) that maximizes the margin

Table S1: Chemical family composition of the 835-molecule reference dataset. Each molecule is assigned to a single family, as recorded in the released dataset. The four hydrocarbon families (paraffins, cyclic paraffins, aromatics, and alkenes) comprise 637 molecules and the three oxygen-containing families (esters, ethers, and aromatic esters) 198.

**Chemical Family**	**Number of Molecules**
Aromatics	256
Cyclic Paraffins	182
Paraffins	152
Esters	103
Ethers	60
Alkenes	47
Aromatic Esters	35
**Total**	**835**

between data points of different classes in a classification problem. With support vector regression, the goal is to find a function that fits the data with as small a deviation as possible within a specified margin.[3] Hyperparameters were selected by random search, sampling 25 configurations per outer fold from the following ranges: regularization parameter $C \in \{0.01, 0.1, 1, 3, 5, 10\}$, epsilon-insensitive loss parameter $\epsilon \in \{0.001, 0.01, 0.1, 0.2\}$, and kernel types {linear, polynomial, radial basis function (RBF), sigmoid}.

Random Forests

Random forests are a widely used ensemble learning method based on aggregating the predictions of multiple decision trees to improve model accuracy and robustness.[4] Unlike a single decision tree, which is prone to overfitting and sensitive to noise, random forests reduce variance by training each tree on a bootstrap sample of the data and introducing randomness in the feature-selection process at each split. This approach leads to improved generalization and stability across a wide range of prediction tasks, making it a strong baseline for both regression and classification problems. Hyperparameters were selected by random search, sampling 25 configurations per outer fold from the following ranges: number of trees `n_estimators` $\in \{100, 200, 300, 500, 1000\}$, maximum tree depth `max_depth` $\in \{$`None`$, 10, 30, 50, 100, 300, 500\}$, minimum number of samples required to split

Table S2: Source method of the property labels used for machine-learning (ML) training, recovered from the source databases: labels reported as direct experimental measurements versus labels obtained by the databases' own correlation, interpolation, or reference property models (see Methods). REFPROP denotes the NIST Reference Fluid Thermodynamic and Transport Properties Database,[1] whose values derive from multiparameter equation-of-state and transport-property correlations fitted to critically evaluated experimental data for well-characterized fluids; the NIST WebBook served a small number of labels from it rather than from direct measurements. The viscosity source method is assigned per label, from the source rows whose converted values resolved that label; labels resolved as the median of duplicate rows with differing source methods are reported as mixed. Fractions are of the labels available for each property; the density and thermal-conductivity class counts at 100 °C differ from those shown by at most one label.

**Property**	**Source method**	**Labels**	**Fraction**
Viscosity (40 °C)	Explicitly experimental	446	55.8%
	Correlation/interpolation (PPDS 9)	207	25.9%
	Correlation/interpolation (ViscosityL)	141	17.6%
	Reference property model (NIST REFPROP)	2	0.3%
	Mixed duplicate sources (median-resolved)	3	0.4%
Viscosity (100 °C)	Explicitly experimental	446	55.5%
	Correlation/interpolation (PPDS 9)	208	25.9%
	Correlation/interpolation (ViscosityL)	140	17.4%
	Reference property model (NIST REFPROP)	2	0.2%
	Mixed duplicate sources (median-resolved)	8	1.0%
Density (40 °C)	Correlation/interpolation (VDNS expansion)	762	99.0%
	Reference property model (NIST REFPROP)	4	0.5%
	Method not recovered	3	0.4%
	Critically evaluated correlation (NIST WTT)	1	0.1%
Thermal cond. (40 °C)	Correlation/interpolation (TransportPolynomial)	768	97.8%
	Method not recovered	11	1.4%
	Reference property model (NIST REFPROP)	5	0.6%
	Critically evaluated correlation (NIST WTT)	1	0.1%

a node `min_samples_split` $\in \{2, 5, 10, 15\}$, minimum number of samples required to be a leaf node `min_samples_leaf` $\in \{1, 2, 4, 6, 8\}$, and maximum number of features considered for splitting `max_features` $\in \{$`'sqrt'`, `'log2'`$\}$.

k-Nearest Neighbors

The *k*-nearest-neighbors (*k*-NN) algorithm is a simple yet effective nonparametric method used for both classification and regression tasks. It makes predictions based on the labels

Table S3: Features included in 3D molecular graphs.

Feature Name	Description
**atomic_number**	Atomic number of the atom
**degree**	Number of directly bonded neighbors
**valence**	Valence of the atom
**formal_charge**	Formal charge assigned to the atom
**aromatic**	Boolean flag indicating aromaticity
**mmff94_partial_charge**	Partial atomic charge from MMFF94
**hybridization**	Hybridization state (e.g., $sp^3 = 3$)
**total_num_h**	Total number of hydrogens bonded to the atom
**in_ring**	Boolean flag for whether atom is in a ring
**chiral_tag**	Chiral tag indicating stereochemistry
**electronegativity**	Pauling electronegativity value
**num_neighbour_C**	Number of directly bonded carbon atoms
**num_neighbour_O**	Number of directly bonded oxygen atoms
**bond_length**	Euclidean distance between bonded atoms
**is_conjugated**	Boolean flag for bond conjugation
**bond_type**	Type of bond (e.g., single = 1)
**bond_order**	Order of the bond (e.g., single = 1)
**bond_stereo**	Bond stereochemistry code
**rotatable**	Boolean flag for rotatable bond
**is_in_ring**	Boolean flag for ring membership

or values of the k most similar data points in the training set, as measured by a distance metric in feature space.[5] It has been used for decades for molecular classification and regression tasks. Hyperparameters were selected by random search, sampling 25 configurations per outer fold from the following ranges: the number of neighbors `n_neighbors` $\in \{2, 5, 7, 9, 15, 21\}$, weighting scheme `weights` $\in$ {`uniform`, `distance`}, distance metric power parameter `p` $\in \{1, 2, 3\}$, and algorithm for neighbor search `algorithm` $\in$ {`auto`, `ball_tree`, `kd_tree`, `brute`} We also varied the leaf size `leaf_size` $\in \{10, 20, 30, 40, 50, 100, 200, 500\}$, which can influence the efficiency of the underlying tree-based search algorithms.

XGBoost

XGBoost (Extreme Gradient Boosting) is a powerful and scalable ML algorithm widely used for regression and classification tasks in cheminformatics.[6–9] It is based on gradient-boosted

decision trees, where an ensemble of weak learners is trained sequentially. Each new tree aims to correct the errors of the previous ensemble by minimizing an overall objective function that combines a loss term and a regularization penalty.[10]

Hyperparameters were selected by random search, sampling 25 configurations per outer fold from the following ranges: number of estimators `n_estimators` $\in \{100, 200, 300, 500, 1000, 2000, 5000\}$, learning rate `lr` $\in \{0.01, 0.05, 0.1, 0.2, 0.5\}$, maximum tree depth `max_depth` $\in \{3, 4, 5, 6, 10, 20, 50, 100\}$, minimum child weight `min_child_weight` $\in \{1, 2, 3, 4, 5, 6\}$, and sampling ratios for both rows `subsample` $\in [0.7, 1.0]$ and columns `colsample_bytree` $\in 0.7, 0.8, 0.9, 1.0$.

Message Passing Neural Networks

Message Passing Neural Networks (MPNNs) are a class of graph neural networks designed for learning on molecular graphs.[11] An MPNN operates on undirected molecular graphs $G = (V, E)$ with node features X_v and edge features E_{km}, and consists of two main phases: a message passing phase and a readout phase. During the message passing phase, information is propagated across the graph using message functions M_t and node update functions U_t to learn intermediate node representations, while the readout phase aggregates these representations using a readout function R to compute a fixed-length feature vector that models the molecular properties of interest. MPNNs are well-suited for molecular property prediction tasks, as they can incorporate both node and edge features to learn chemically meaningful representations, and versions of this architecture, such as the directed-MPNN, have been shown in literature to provide superior performance to other deep learning architectures.[12–14] Hyperparameters were selected by random search, sampling 25 configurations per outer fold from the following ranges: learning rate `lr` sampled log-uniformly from $[10^{-4}, 10^{-2}]$, batch size `batch_size` $\in \{8, 16, 32\}$, optimizer `optimizer` $\in \{$`Adam`, `RMSProp`$\}$, number of message-passing layers `num_layers` $\in \{1, 2, 3, 4, 5\}$, hidden-layer size `hidden_dim` $\in \{16, 32, 64, 128\}$, and dropout `dropout` $\in \{0, 0.1, 0.2, 0.3\}$; all activations use the rectified linear unit (ReLU) function.

Graph Attention Networks

Graph attention networks (GATs) are a type of graph neural network that incorporates attention to weight the importance of neighboring nodes during message passing.[15] Unlike traditional graph convolutional models, which aggregate information from neighbors with equal or fixed weights, GATs learn attention coefficients that allow the model to focus on the most relevant local substructures for a given prediction task. This makes them very effective for learning on molecular graphs, where the significance of atomic neighbors may vary based on chemical context. Hyperparameters were selected by random search, sampling 25 configurations per outer fold from the following ranges: learning rate `lr` sampled log-uniformly from $[10^{-4}, 10^{-2}]$, batch size `batch_size` $\in \{8, 16, 32\}$, optimizer `optimizer` $\in \{\texttt{Adam}, \texttt{RMSProp}\}$, number of hidden units `hidden_dim` $\in \{16, 32, 64, 128\}$, number of attention heads `num_heads` $\in \{1, 2, 4\}$, number of layers `num_layers` $\in \{1, 2, 3, 4, 5\}$, and dropout `dropout` $\in \{0, 0.1, 0.2, 0.3\}$; all activations use ReLU.

Convolutional Neural Networks

Convolutional neural networks (CNNs) are a class of deep-learning models originally designed for image and spatial data,[16] but have also been applied to sequential and structured data such as simplified molecular-input line-entry system (SMILES) strings or molecular fingerprints.[17] CNNs leverage convolutional layers to detect local patterns through sliding filters, enabling the model to learn hierarchical feature representations that are useful for capturing spatial or sequential dependencies.[16] In molecular property prediction, one-dimensional (1D) CNNs are often applied to representations such as SMILES or self-referencing embedded strings (SELFIES), where local substructure patterns are informative for property prediction.[18] Hyperparameters were selected by random search, sampling 25 configurations per outer fold from the following ranges: optimizer type `optimizer` $\in \{\texttt{Adam}, \texttt{RMSProp}\}$, hidden-layer size `hidden_dim` $\in \{16, 32, 64, 128\}$, number of convolutional layers `num_layers` $\in \{1, 2, 3, 4, 5\}$, number of filters per layer `filters` $\in \{32, 64, 128\}$,

and kernel size `kernel_size` $\in \{3, 5, 7\}$. We also varied the batch size `batch_size` $\in \{16, 32, 64, 128\}$ to explore its effect on training stability and generalization. The learning rate was fixed at 0.001 and all activations are ReLU.

Multilayer Perceptron

Multilayer perceptrons (MLPs) are a class of feedforward artificial neural networks that have been widely applied to molecular property prediction in chemistry.[19–21] An MLP consists of an input layer, one or more hidden layers, and an output layer, where each neuron performs a weighted sum of its inputs followed by a nonlinear activation function. These nonlinear transformations enable MLPs to capture complex, high-dimensional relationships between molecular descriptors and target properties.

Network parameters are optimized by minimizing a task-dependent loss function using gradient-based optimization algorithms such as stochastic gradient descent or Adam, with gradients computed via backpropagation.[22,23] Owing to their flexibility and ability to model nonlinear feature interactions, MLPs are commonly employed as baseline models in molecular property prediction tasks, particularly for moderate-sized datasets.[8]

Hyperparameters were selected by random search, sampling 25 configurations per outer fold from the following ranges: optimizer type `optimizer` $\in \{\texttt{Adam}, \texttt{RMSProp}\}$, hidden-layer dimensionality `hidden_dim` $\in \{16, 32, 64, 128\}$, number of hidden layers `num_layers` $\in \{1, 2, 3, 4, 5\}$, and batch size `batch_size` $\in \{8, 16, 32, 64\}$; the learning rate was fixed at 0.001 and all activations use ReLU.

Transformers

The transformer architecture introduced by Vaswani et al.[24] was originally developed for processing sequential data, in an attempt to overcome the vanishing gradient problem present when training recurrent neural networks.[25] It uses a self-attention mechanism to compute pairwise dependency values between all input tokens, enabling efficient modeling of both

short-range and long-range interactions.

Hyperparameters were selected by random search, sampling 25 configurations per outer fold from the following ranges: $\texttt{batch\_size} \in \{8, 16, 32\}$, $\texttt{optimizer} \in \{\texttt{Adam}, \texttt{RMSProp}\}$, $\texttt{hidden\_dim} \in \{16, 32, 64\}$, number of transformer blocks $\texttt{num\_blocks} \in \{1, 2, 3, 4, 5\}$, and number of self-attention heads $\texttt{num\_heads} \in \{1, 2, 4\}$; the learning rate was fixed at 0.001 and all activations use ReLU.

ChemBERTa

ChemBERTa is a transformer-based model built on the RoBERTa architecture and pre-trained on large-scale chemical language datasets such as SMILES.[26–28] This study used the ChemBERTa-77M-MTR checkpoint, pretrained on 77 million canonicalized SMILES strings with a multitask regression objective over 200 RDKit-computed molecular properties. Two configurations were evaluated under the shared locked-fold protocol. **Fine-tuned SMILES:** the full pretrained encoder is fine-tuned end-to-end for each property and temperature, with a linear regression head applied to the attention-mask-weighted mean-pooled token representations. All encoder weights are trainable; optimization uses AdamW for up to 30 epochs with early stopping (patience 4) on the fold's validation partition. Hyperparameters were selected by random search over 25 configurations per outer fold, with the learning rate sampled log-uniformly from 10^{-6} to 10^{-4} and batch size from {8, 16, 32}. **Frozen embeddings + MLP:** the pretrained encoder is used as a fixed feature extractor with no fine-tuning: each molecule's SMILES is encoded once and the attention-mask-weighted mean-pooled output is stored as a fixed-length embedding vector. An MLP head is then trained on these frozen vectors, with random search over the optimizer (Adam or RMSProp), batch size $\in \{8, 16, 32, 64\}$, hidden-layer size $\in \{16, 32, 64, 128\}$, and number of layers $\in \{1, \ldots, 5\}$; the learning rate was fixed at 0.001. The same frozen embeddings were also used as the input representation for an XGBoost model ("XGBoost + ChemBERTa embeddings" in the benchmark tables).

Software versions

All ML models were trained with Python 3.10.20 using the following package versions: numpy 1.26.4, pandas 2.3.3, scikit-learn 1.7.2, scipy 1.15.2, xgboost 3.2.0, RDKit 2025.09.5, Mordred 1.2.0, SELFIES 2.1.1, umap-learn 0.5.11, PyTorch 2.10.0, PyTorch Geometric 2.7.0, and HuggingFace Transformers 5.2.0. A conda environment specification pinning the core versions is provided with the released code, together with the exact command that installs the pinned PyTorch, PyTorch Geometric, and HuggingFace Transformers versions used for the graph and transformer models; these are installed as a separate step because their wheels depend on the local CUDA build (see Data and Software Availability in the main text). Molecular-dynamics (MD) simulations used the Large-scale Atomic/Molecular Massively Parallel Simulator (LAMMPS; 29 August 2024 release), and the analysis used numpy/scipy as above.

Results and Discussion

Optimization of Molecular Representations

To optimize the molecular representations used in this study, a systematic grid search was conducted for both embedding and fingerprint-based features. For embeddings, the effect of dimensionality (10–750 features) was evaluated using principal component analysis (PCA) and uniform manifold approximation and projection (UMAP), while for Morgan fingerprints, bit sizes (256–4096) and radii (1–10) were explored. These searches used a single 80:10:10 train/validation/test split rather than the locked folds of the benchmark, and were used only to fix the representation configurations (100-dimensional PCA embeddings; unhashed radius-8 Morgan fingerprints) carried into the benchmark, and no performance claims in the main text are based on them. Detailed results and performance trends for these optimization studies are provided below.

Embeddings

Figure S2 shows the effect of different embedding methods and dimensionality on XGBoost prediction of the thermophysical properties. Embedding dimensionalities from 10 to 750 were evaluated for both PCA and UMAP to assess performance across a broad range of feature compression levels, using 5-fold cross-validation and an 80:10:10 train:validation:test split. This range aimed to explore the tradeoff of maintaining the diversity and distribution of chemical and physical characteristics represented by the molecular descriptors within the dataset and minimizing the risk of overfitting due to redundant or noisy features. A prior study found that as few as 12 molecular descriptors optimized by recursive feature elimination (RFE) and validated by PCA were sufficient to accurately predict the viscosity of binary mixtures with an R^2 of 0.98 and a mean-squared error of 1.5.[29] As shown by Figure S2, there is some variance in the optimal embedding dimensionality across different properties and temperatures, but overall, PCA embeddings consistently outperformed UMAP across

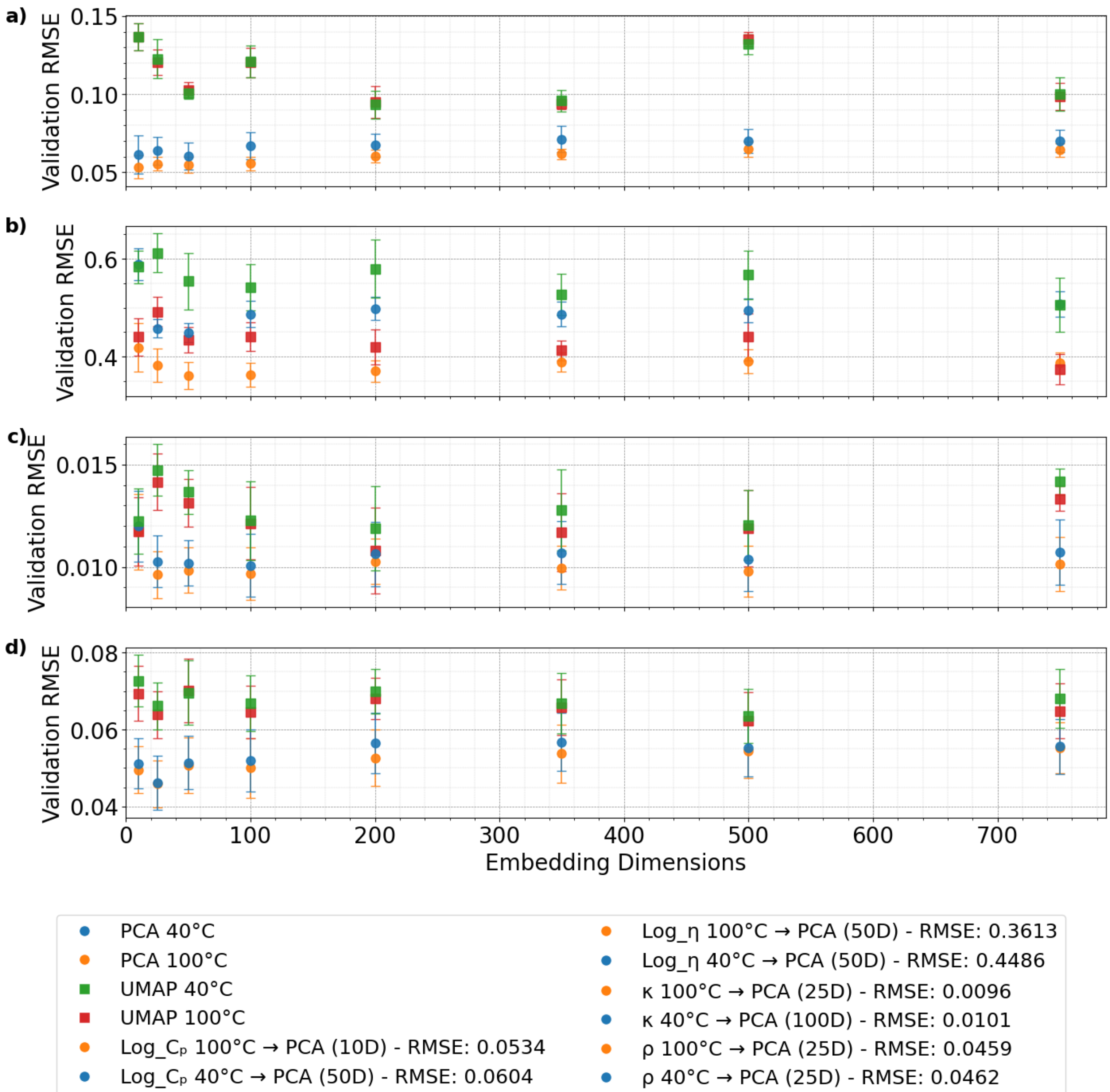

Figure S2: Effect of embedding method and dimensionality on XGBoost prediction of (a) log heat capacity, $\log C_p$ (retained from an early screening stage and not analyzed further), (b) log viscosity, $\ln(\eta/\mathrm{mPa\,s})$, (c) thermal conductivity, κ, and (d) density, ρ. Legends give the best method–dimension combination per property; error bars are one standard deviation over 5-fold cross-validation.

most properties and dimensionalities. This variance suggests that the minimum number of descriptors required to optimize performance is strongly dependent on the property. However, the best-performing dimensionality for each property never exceeded 100 dimensions.

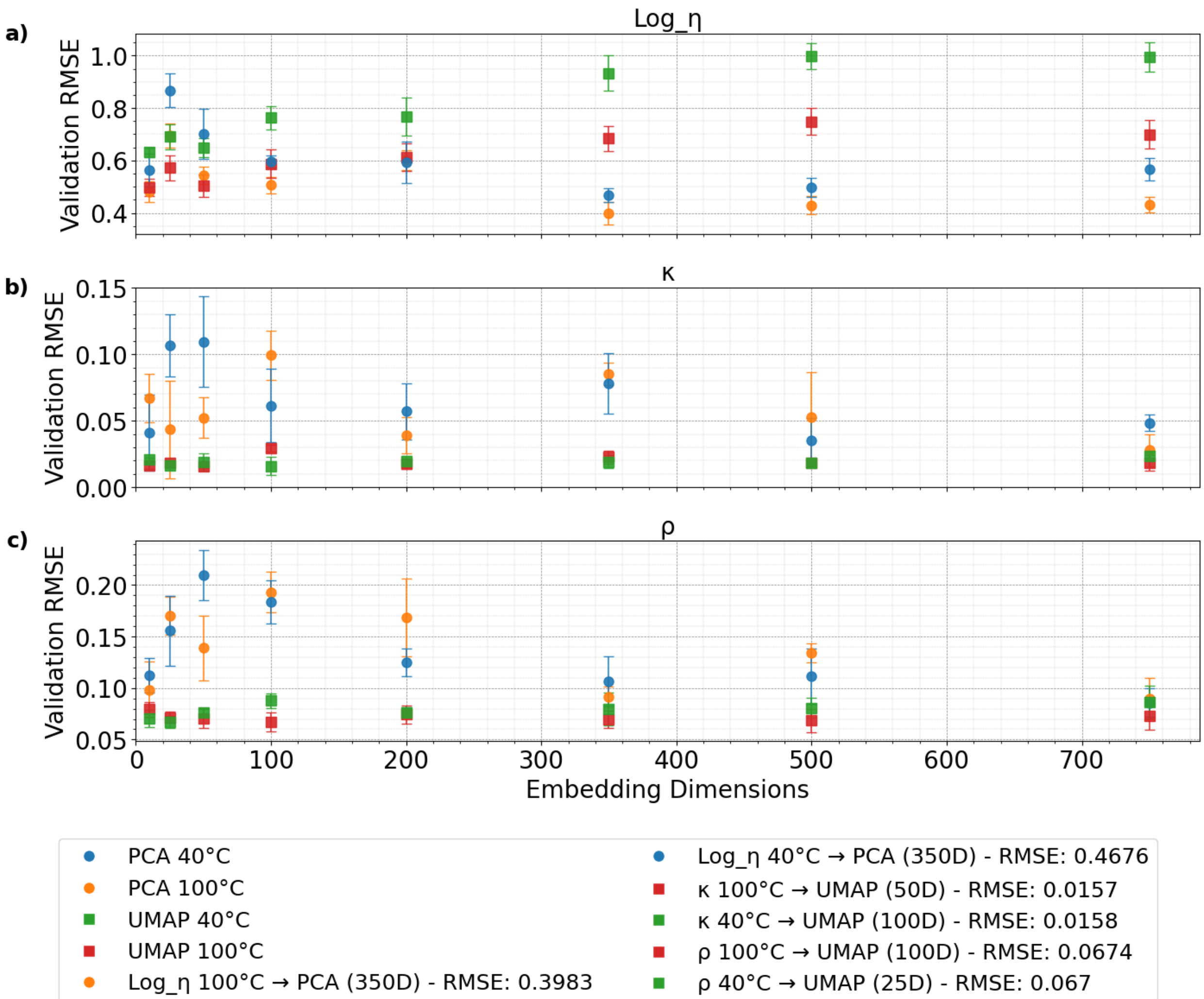

Figure S3: Effect of embedding method and dimensionality on MLP prediction of (a) log viscosity, $\ln(\eta/\mathrm{mPa\,s})$, (b) thermal conductivity, κ, and (c) density, ρ. Legends give the best method–dimension combination per property; error bars are one standard deviation over 5-fold cross-validation.

This may reflect greater nonlinearity in the relationship between molecular structure and these properties.

PCA's linear assumption, with dimensionality reduction achieved by identifying axes of maximal global variance, can create a strong synergy with models such as XGBoost, which, while not linear themselves, rely on axis-aligned splits and benefit from input features that expose monotonic relationships with the target variable.[30] The linear transformations produced by PCA often enhance the signal in dominant variance directions, most likely

making the resulting embeddings more compatible for tree-based models. UMAP is designed to preserve local neighborhood structure and optimize manifold (lower-dimensional space) topology for visualization or clustering tasks, rather than to maintain global variance.[31] This can result in embeddings that obscure relationships between features and target variables, reducing compatibility with tree-based models.

To investigate whether the superior performance of PCA is due to synergistic effects with XGBoost, an MLP was trained with the same range of embeddings. As with the XGBoost models, most of the properties were best predicted with embedding sizes of 100 dimensions or less, except for viscosity at the two temperatures, which needed much greater dimensionality to maximize the predictive performance of the MLP. Interestingly, while PCA often outperforms UMAP when used with tree-based models like XGBoost, we find that UMAP embeddings yield superior performance when used with MLPs, for 4 of the 6 properties (Figure S3). This may be because MLPs can recover the nonlinear patterns embedded in the locally structured manifolds UMAP creates, compensating for the global distortions introduced by UMAP and making it a more compatible embedding method for MLPs and other deep models, especially as it is likely that the true structure–property relationships are nonlinear and high-dimensional. Despite this, we observed that overall cross-validation root-mean-square error (RMSE) values were consistently lower for XGBoost than for MLP across all embeddings and properties, suggesting that XGBoost remains more robust for embedding-based molecular representations.

Table S4: Best-performing validation RMSE and corresponding embedding dimension for each property using PCA and UMAP embeddings with XGBoost and MLP models. Values are shown as *RMSE (dimension)*, and the lowest RMSE per property is bolded.

**Property**	**PCA XGBoost**	**PCA MLP**	**UMAP XGBoost**	**UMAP MLP**
Density 100°C	**0.046 (25)**	0.090 (750)	0.062 (500)	0.067 (100)
Density 40°C	**0.046 (25)**	0.086 (750)	0.064 (500)	0.067 (25)
Viscosity 100 °C (ln)	**0.361 (50)**	0.398 (350)	0.374 (750)	0.497 (10)
Viscosity 40 °C (ln)	**0.449 (50)**	0.468 (350)	0.505 (750)	0.631 (10)
Thermal Conductivity 100°C	**0.010 (25)**	0.028 (750)	0.011 (200)	0.016 (50)
Thermal Conductivity 40°C	**0.010 (100)**	0.035 (500)	0.012 (200)	0.016 (100)

Table S4 summarizes the best-performing embedding dimension and validation RMSE for each property and the best-performing model trained during cross-validation, for the XGBoost and MLP models and the two dimensionality-reduction methods. The XGBoost models trained with PCA embeddings consistently outperformed the other three combinations. We also note that many more dimensions are required to maximize the performance of models trained on the UMAP embeddings than of those trained on the PCA embeddings for the XGBoost models, and vice versa for the MLP models, further highlighting the incompatibility of certain dimensionality-reduction techniques with certain model types. However, the use of UMAP embeddings did not consistently lead to significantly improved prediction performance by the MLP models compared to the models trained on PCA embeddings. Based on these findings, in the full benchmarking studies, we use PCA embeddings of 100 dimensions for all of the models.

Fingerprints

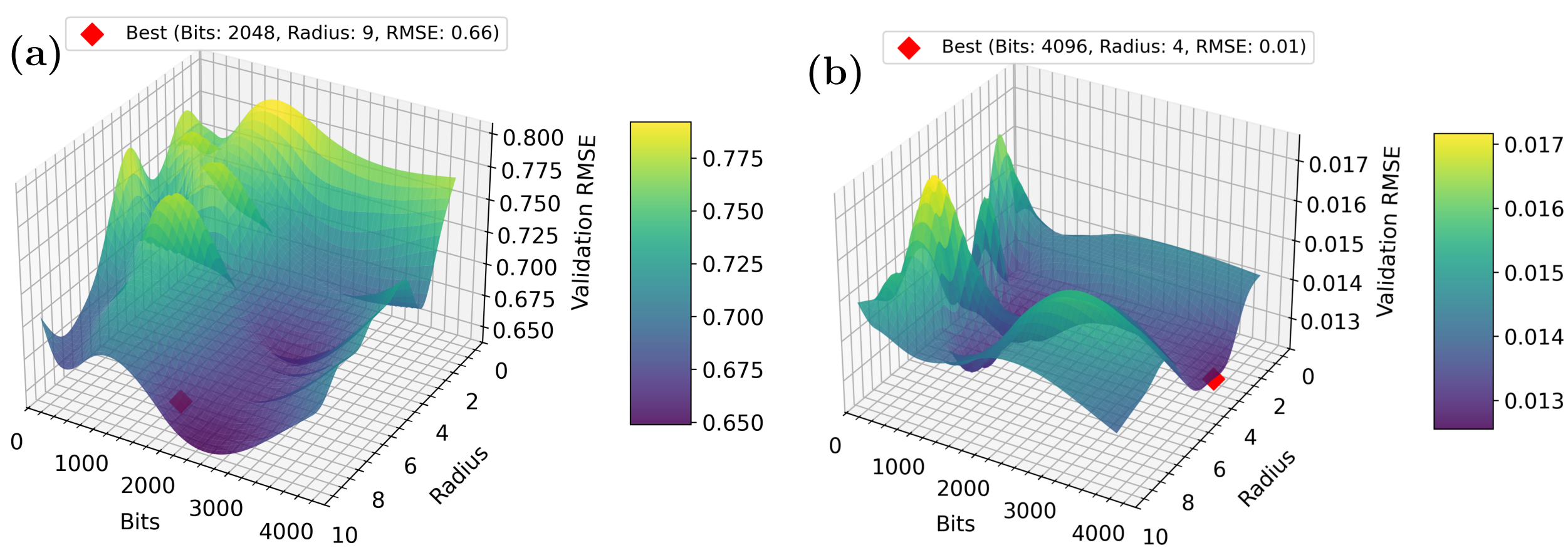

Figure S4: Validation RMSE surface plots for XGBoost across bit size and radius for (a) Viscosity and (b) Thermal Conductivity at 40°C.

We evaluated how extended-connectivity fingerprint (ECFP) generation influences molecular-property prediction using ML. We generated binary Morgan fingerprints for each molecule across a range of fingerprint radii (1–10) and bit sizes (256–4096). Varying bit sizes from 256

to 4096 allowed us to investigate how bit density and collision frequency scale with fingerprint size, which becomes increasingly relevant in large molecules where many substructures must be uniquely encoded. XGBoost and MLP models were trained to predict the six properties using an 80:10:10 train/validation/test split, again with calculation of 5-fold cross-validation RMSE and validation RMSE.

Table S5: Best-performing ECFP configurations by model and property.

Model	Property	Bits	Radius	Cross Val. RMSE	Test RMSE	Bit Collisions
MLP	Density 100°C	1024	1	0.0662	0.0459	112
XGBoost	Density 100°C	4096	2	0.0575	0.0411	697
MLP	Density 40°C	2048	1	0.0645	0.0509	108
XGBoost	Density 40°C	4096	2	0.0588	0.0426	697
MLP	T. Cond. 100°C	4096	4	0.0165	0.0141	2513
XGBoost	T. Cond. 100°C	4096	4	0.0149	0.0120	2513
MLP	T. Cond. 40°C	2048	4	0.0180	0.0146	122
XGBoost	T. Cond. 40°C	4096	4	0.0157	0.0128	2513
MLP	Viscosity 100°C	1024	9	0.4752	0.4480	1024
XGBoost	Viscosity 100°C	1024	9	0.4767	0.4075	512
MLP	Viscosity 40°C	1024	9	0.6301	0.6563	1024
XGBoost	Viscosity 40°C	2048	9	0.6528	0.6639	2046

Table S5 shows the best-performing ECFP configurations for each property for the trained MLP and XGBoost models. We observed that, at a fixed bit size, the bit collision rate rose with fingerprint radius. This is expected, as larger radii capture more extended atom environments, introducing greater substructure diversity that must share the same hashing space. Despite this rise in collisions, both XGBoost and MLP models exhibited improved predictive performance with larger radii, as larger environments encode more diverse and informative chemical substructures. Increasing the bit size reduces the probability of collision per bit by expanding the hashing space, mitigating this downside of larger radii, and predictive performance accordingly also improved with bit size.

As shown by Table S5, the XGBoost models again outperform all of the MLP models. We find that the optimal fingerprint configuration varies by property, most likely because different molecular properties are influenced by different structural features and interaction

patterns, as illustrated by Fig. S4. Thermal conductivity and density are often governed by local atomic environments such as atomic packing and short-range intermolecular forces, which can be more effectively captured by fingerprints with smaller radii.[32] However, viscosity depends more heavily on global molecular features, such as overall molecular size, shape, branching, and flexibility, which influence the distribution of vibrational modes or resistance to flow.[32] These more complex features are better represented by larger radii fingerprints that encode extended substructures and longer-range connectivity.

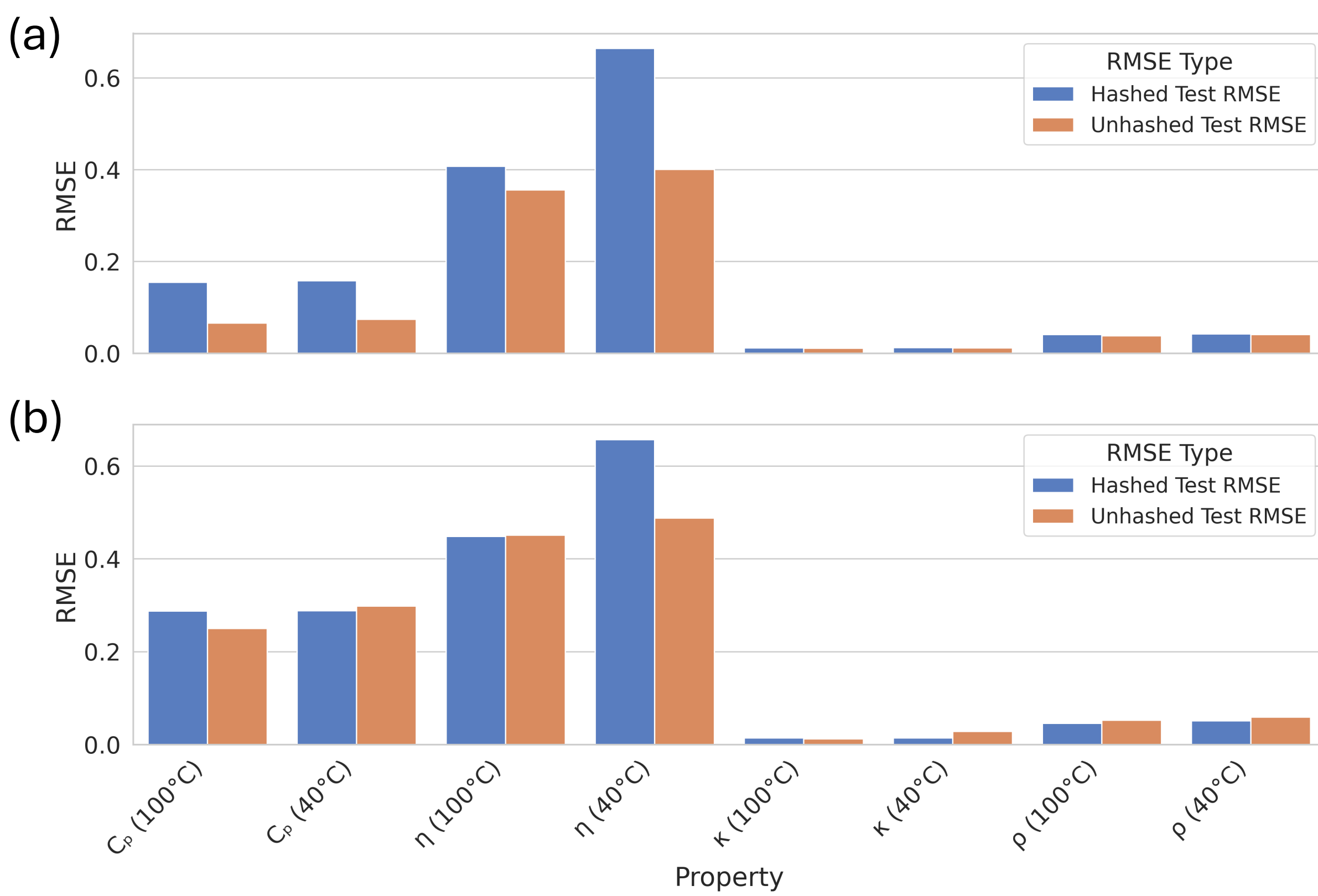

Figure S5: Test RMSE for the best-performing hashed fingerprint configurations retrained with unhashed fingerprints at the same radius, for (a) XGBoost and (b) MLP. Blue bars are hashed and orange bars unhashed fingerprints.

Hashed ECFP fingerprints limit interpretability due to bit collisions, which prevent direct tracing of model predictions to specific chemical features. To address this, unhashed Morgan fingerprints were used with a fixed radius, avoiding compression of substructure identifiers into a fixed-length bit vector. This approach eliminates bit collisions and ensures that each

substructure retains a unique representation in the feature space.

Unhashed fingerprints present the tradeoff of potentially leading to very high-dimensional and sparse feature spaces, especially for large or diverse molecular datasets, increasing memory consumption and degrading performance. However, as shown in Fig. S5, models trained on unhashed fingerprints achieved comparable or better performance on the test set than those trained on hashed fingerprints. This improvement is likely due to the complete removal of collisions, allowing the model to treat each chemical environment as a distinct input and more effectively learn structure–property relationships, compensating for any sparsity in the representation. Based on these results, unhashed fingerprints with a radius of 8 were used in all benchmarking studies to improve the prediction of complex properties such as viscosity.

Best Representations and ML Performance

Table S6: Benchmark of the ML model–representation combinations: test RMSE and, in parentheses, R^2, each a mean over the five locked outer folds. Viscosity RMSE is on $\ln(\eta/\mathrm{mPa\,s})$ and uses the dynamic-viscosity dataset (799 labels at 40 °C and 804 at 100 °C); density is in $\mathrm{g\,cm^{-3}}$ and thermal conductivity in $\mathrm{W\,m^{-1}\,K^{-1}}$. Rows are ordered by overall rank (z-score-normalized RMSE averaged over columns); the best value in each column is in bold. Between-fold standard deviations accompany the released results, and per-fold metrics can be recomputed from the released out-of-fold (OOF) predictions.

	Viscosity, η		Density, ρ		Thermal cond., κ	
Model + representation	40 °C	100 °C	40 °C	100 °C	40 °C	100 °C
XGBoost + descriptors	**0.353 (0.86)**	**0.298 (0.83)**	0.048 (0.76)	0.048 (0.74)	0.010 (0.88)	0.010 (0.87)
SVM + PCA embeddings	0.383 (0.84)	0.314 (0.81)	0.051 (0.73)	0.049 (0.73)	0.010 (0.88)	**0.008 (0.91)**
Random forest + descriptors	0.370 (0.85)	0.313 (0.81)	**0.047 (0.77)**	**0.046 (0.77)**	0.011 (0.85)	0.011 (0.85)
MLP + ChemBERTa embeddings	0.411 (0.81)	0.337 (0.78)	0.051 (0.73)	0.047 (0.75)	**0.009 (0.89)**	0.009 (0.88)
MLP + PCA embeddings	0.432 (0.79)	0.366 (0.73)	0.054 (0.69)	0.051 (0.71)	0.010 (0.88)	0.009 (0.89)
XGBoost + ChemBERTa embeddings	0.406 (0.82)	0.318 (0.80)	0.050 (0.74)	0.050 (0.72)	0.012 (0.82)	0.011 (0.84)
XGBoost + PCA embeddings	0.386 (0.84)	0.321 (0.80)	0.051 (0.72)	0.052 (0.70)	0.012 (0.84)	0.011 (0.84)
CNN + SMILES	0.453 (0.78)	0.354 (0.75)	0.052 (0.72)	0.051 (0.72)	0.011 (0.84)	0.012 (0.81)
k-NN + descriptors	0.444 (0.79)	0.356 (0.75)	0.051 (0.72)	0.050 (0.72)	0.014 (0.78)	0.013 (0.78)
k-NN + PCA embeddings	0.453 (0.78)	0.370 (0.73)	0.053 (0.70)	0.052 (0.70)	0.013 (0.80)	0.012 (0.80)
SVM + descriptors	0.381 (0.84)	0.315 (0.80)	0.053 (0.70)	0.052 (0.70)	0.015 (0.73)	0.014 (0.72)
CNN + SELFIES	0.445 (0.78)	0.361 (0.74)	0.055 (0.68)	0.052 (0.70)	0.014 (0.78)	0.013 (0.78)
Transformer + SMILES	0.471 (0.76)	0.372 (0.73)	0.050 (0.73)	0.052 (0.70)	0.016 (0.70)	0.015 (0.68)
MPNN + 3D graph	0.634 (0.56)	0.468 (0.57)	0.052 (0.72)	0.052 (0.70)	0.014 (0.75)	0.014 (0.73)
Transformer + SELFIES	0.463 (0.77)	0.380 (0.72)	0.051 (0.73)	0.051 (0.71)	0.017 (0.64)	0.017 (0.61)
Random forest + PCA embeddings	0.524 (0.70)	0.412 (0.67)	0.057 (0.65)	0.055 (0.66)	0.016 (0.70)	0.015 (0.70)
XGBoost + fingerprints	0.672 (0.51)	0.545 (0.42)	0.057 (0.66)	0.055 (0.66)	0.014 (0.75)	0.013 (0.77)
SVM + fingerprints	0.651 (0.54)	0.503 (0.50)	0.060 (0.62)	0.059 (0.61)	0.014 (0.75)	0.014 (0.76)
MLP + fingerprints	0.679 (0.50)	0.524 (0.46)	0.062 (0.60)	0.060 (0.61)	0.015 (0.75)	0.014 (0.74)
ChemBERTa (fine-tuned SMILES)	0.372 (0.85)	0.313 (0.81)	0.067 (0.51)	0.067 (0.50)	0.017 (0.63)	0.018 (0.48)
Transformer + PCA embeddings	0.534 (0.69)	0.391 (0.70)	0.059 (0.64)	0.055 (0.67)	0.019 (0.58)	0.019 (0.53)
Random forest + fingerprints	0.750 (0.39)	0.579 (0.34)	0.060 (0.63)	0.059 (0.62)	0.015 (0.73)	0.014 (0.73)
MLP + SMILES	0.512 (0.71)	0.416 (0.66)	0.062 (0.60)	0.063 (0.55)	0.018 (0.61)	0.019 (0.56)
GAT + 3D graph	0.784 (0.33)	0.631 (0.22)	0.057 (0.66)	0.056 (0.66)	0.015 (0.71)	0.017 (0.61)
MLP + SELFIES	0.540 (0.68)	0.418 (0.66)	0.066 (0.54)	0.063 (0.56)	0.020 (0.54)	0.019 (0.55)
k-NN + fingerprints	1.140 (-0.41)	0.830 (-0.36)	0.090 (0.13)	0.091 (0.06)	0.020 (0.54)	0.019 (0.52)

Table S7: Label-provenance sensitivity analysis for viscosity. The XGBoost + descriptor models were retrained with the same locked outer folds and the same selected hyperparameters as in the main benchmark, either on all available viscosity labels or only on labels recovered as explicitly experimental, and both were evaluated on the explicitly experimental labels of each untouched outer test fold. Values are mean ± between-fold standard deviation of the test RMSE on $\ln(\eta/\mathrm{mPa\,s})$ over the five folds.

Training labels	Molecules	RMSE 40 °C	RMSE 100 °C
All available labels	≈640	0.272 ± 0.024	0.225 ± 0.065
Explicitly experimental only	≈356	0.277 ± 0.034	0.209 ± 0.055

Out-of-Distribution (OOD) Study

Table S8: OOD test data with different atoms present in structures compared to training data.

**Name**	**Test Property**	**Viscosity 40°C**	**Viscosity 100°C**	**Viscosity Prediction**
Tri-Cresyl Phosphate	Different Atom	30.8	4.2	ViscosityL
diheptylamine	Different Atom	1.8	0.9	Landolt–Börnstein
triisopentylamine	Different Atom	1.7	0.7	Landolt–Börnstein
trihexylamine	Different Atom	2.8	1.8	Landolt–Börnstein
triheptylamine	Different Atom	4.0	1.3	Landolt–Börnstein
trioctylamine	Different Atom	6.0	1.7	Landolt–Börnstein
N-butyldodecanamide	Different Atom	26.0	6.7	ViscosityL
N-cyclohexyllinoleamide	Different Atom	110.0	22.0	PPDS 9
N,N-dimethyloleamide	Different Atom	11.0	3.5	ViscosityL
N-isobutyl-3,3-dipropylheptanamide	Different Atom	41.0	11.0	PPDS 9
N,N-dihexyloctanamide	Different Atom	11.0	3.4	ViscosityL
diisobutyl disulfide	Different Atom	1.6	0.8	PPDS 9
octyl disulfide	Different Atom	3.6	1.6	ViscosityL
didecyl disulfide	Different Atom	5.5	2.2	ViscosityL
diphenyl sulfide	Different Atom	10.0	2.8	ViscosityL
n-dodecyl sulfide	Different Atom	5.4	2.1	ViscosityL
1,6-bis(2-ethylhexylthio)hexane	Different Atom	14.0	4.4	ViscosityL
4,8-dithiadodecane	Different Atom	1.9	1.0	PPDS 9
4-thiahenicosane	Different Atom	3.6	1.6	ViscosityL
4-thiahexadecane	Different Atom	2.1	1.0	PPDS 9

Table S9: OOD test data with higher viscosities compared to training data.

**Name**	**Test Property**	**Viscosity 40°C**	**Viscosity 100°C**	**Viscosity Prediction**
pentaerythritol tetrakis-(2-ethylbutanoate)	High Viscosity	61.4	6.5	Landolt–Börnstein
12-benzoyloxy-octadecanoic acid phenyl ester	High Viscosity	304.6	29.4	Landolt–Börnstein
pentaerythritol dihexanoate dicyclohexanecarboxylate	High Viscosity	99.9	10.0	Landolt–Börnstein
9,10,12,13-tetrakis-(propionyloxy)-octadecanoic acid methyl ester	High Viscosity	72.0	7.7	Landolt–Börnstein
6-(1-butyl-octadecyl)-1,2,3,4-tetrahydro-naphthalene	High Viscosity	51.9	7.1	Landolt–Börnstein
dipentaerythritol hexabutanoate	High Viscosity	64.0	8.8	Landolt–Börnstein
4-(1-butyl-octadecyl)-bicyclohexyl	High Viscosity	76.1	9.0	Landolt–Börnstein
5-biphenyl-4-yl-hexacos-4-ene	High Viscosity	66.0	9.0	Landolt–Börnstein
1-naphthalen-2-yl-1,2-dibutyl-eicosane	High Viscosity	69.0	8.6	Landolt–Börnstein
ethylene glycol bis-(12-acetoxy-stearate)	High Viscosity	84.0	12.0	Landolt–Börnstein
pentanedioic acid bis-(2,2,3,3,4,4,5,5-octafluoropentyl) ester	Different Atom	13.5	2.2	Landolt–Börnstein
2,2,4,15,17,17-hexamethyl-7,12-bis-(3,5,5-trimethylhexyl)-octadecane	High Viscosity	104.0	8.3	Landolt–Börnstein
propylene glycol bis-(12-acetoxy-stearate)	High Viscosity	85.0	12.0	Landolt–Börnstein
trimethylene glycol bis-(12-acetoxy-stearate)	High Viscosity	90.0	13.0	Landolt–Börnstein
diethylene glycol bis-(12-acetoxy-stearate)	High Viscosity	93.0	13.0	Landolt–Börnstein
7,16-dimethyl-9,11,12,14-tetrahexyl-docosane	High Viscosity	80.0	8.0	Landolt–Börnstein
1,10-bis-(naphth-1-yl)-decane	High Viscosity	226.0	11.0	Landolt–Börnstein
1,10-bis-(decahydro-naphth-1-yl)-decane	High Viscosity	145.0	10.9	Landolt–Börnstein
1-cyclohexyl-4-decahydro-naphth-1-yl-tetradecane	High Viscosity	109.0	8.0	Landolt–Börnstein
5-(1-phenethyl-tetradecyl)-1,2,3,4-tetrahydro-naphthalene	High Viscosity	63.1	16.4	Landolt–Börnstein

Table S10: Per-molecule discrimination of OOF error by each signal. r_s is the Spearman correlation between the raw signal and the absolute error in $\ln(\eta/\mathrm{mPa\,s})$. The area under the receiver operating characteristic curve (AUC) is the probability that a random molecule from the signal's top decile has a larger absolute error than a random molecule from the remaining 90%; 0.5 corresponds to no information. Ratio is the median absolute error of the top decile over that of the remainder.

	40 °C			100 °C		
Signal	r_s	AUC	ratio	r_s	AUC	ratio
Mahalanobis	0.33	0.66	1.9	0.41	0.72	2.3
k-NN	0.36	0.63	1.9	0.42	0.75	2.5
Tanimoto	0.30	0.63	1.6	0.27	0.59	1.7
Cross-fold disagreement	0.27	0.63	1.7	0.36	0.75	2.7

Table S11: OOD-set RMSE in $\ln(\eta/\mathrm{mPa\,s})$ by category and temperature.

Category	n	40 °C	100 °C
Heteroatom	21	0.77	0.71
High viscosity	19	1.25	0.60

Molecular Dynamics Case Study

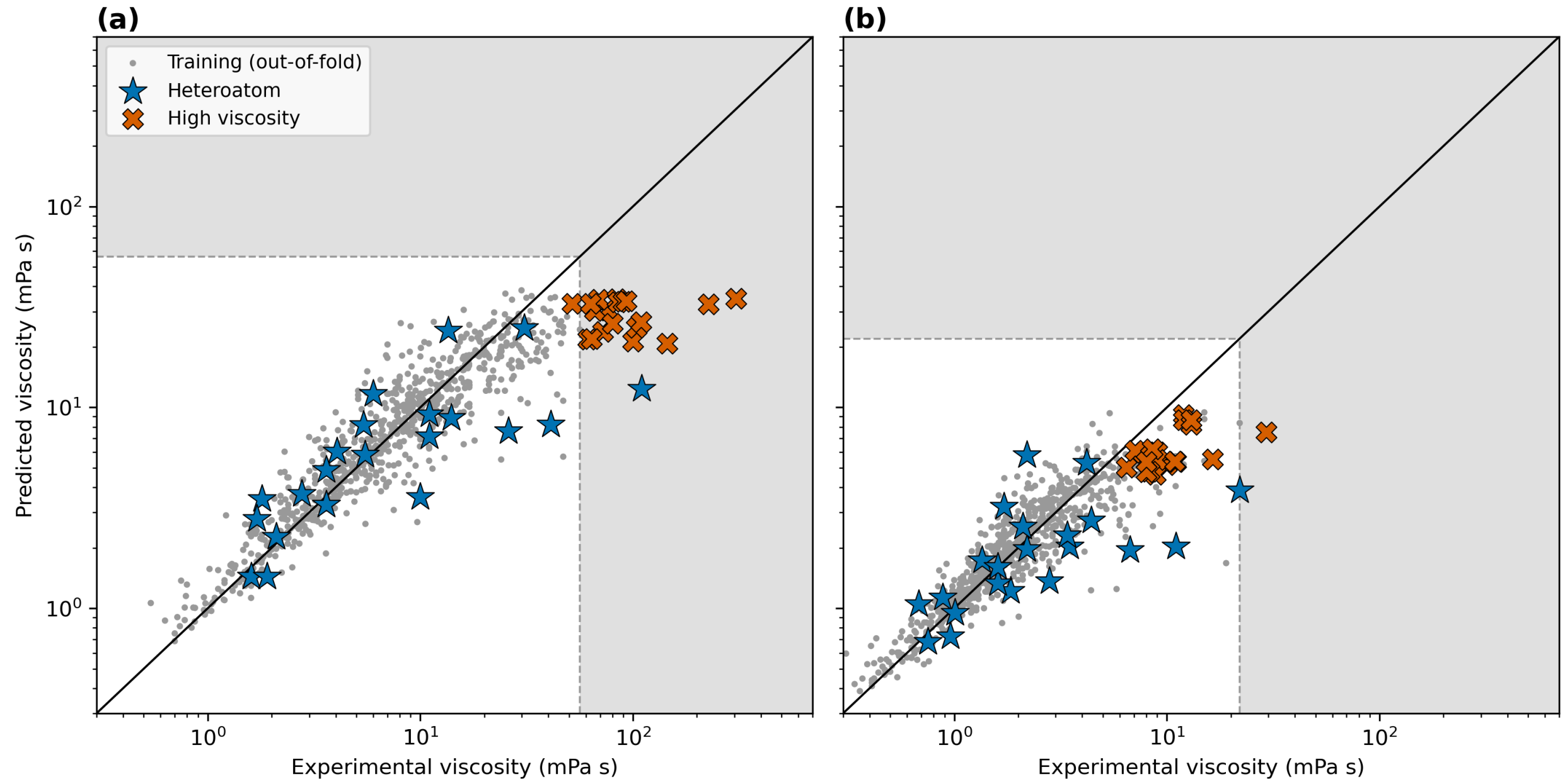

Figure S6: Predicted against experimental viscosity for the 40 OOD molecules at (a) 40 °C and (b) 100 °C, with the OOF training predictions (gray) for context and the unity line in black. The unshaded region is the range spanned by the training labels; the shaded region lies beyond the largest training label on either axis, 56.3 mPa s at 40 °C and 22.0 mPa s at 100 °C. One of the 19 high-viscosity molecules falls inside the training range at 40 °C, against 18 of 19 at 100 °C.

Table S12: Out-of-distribution (OOD) molecules flagged at 40 °C, by category. Signal: molecules beyond the 90th percentile of the training distribution of that signal. Two-axis: molecules beyond the 90th percentile of the signal or with a predicted viscosity above the 90th percentile of the training labels. The final row gives the predicted-value flag alone.

	Heteroatom ($n = 21$)		High viscosity ($n = 19$)	
Signal	signal	two-axis	signal	two-axis
Mahalanobis	21	21	13	19
k-NN	17	17	14	19
Tanimoto	5	5	0	14
Cross-fold disagreement	1	3	2	15
Predicted value alone	2		14	

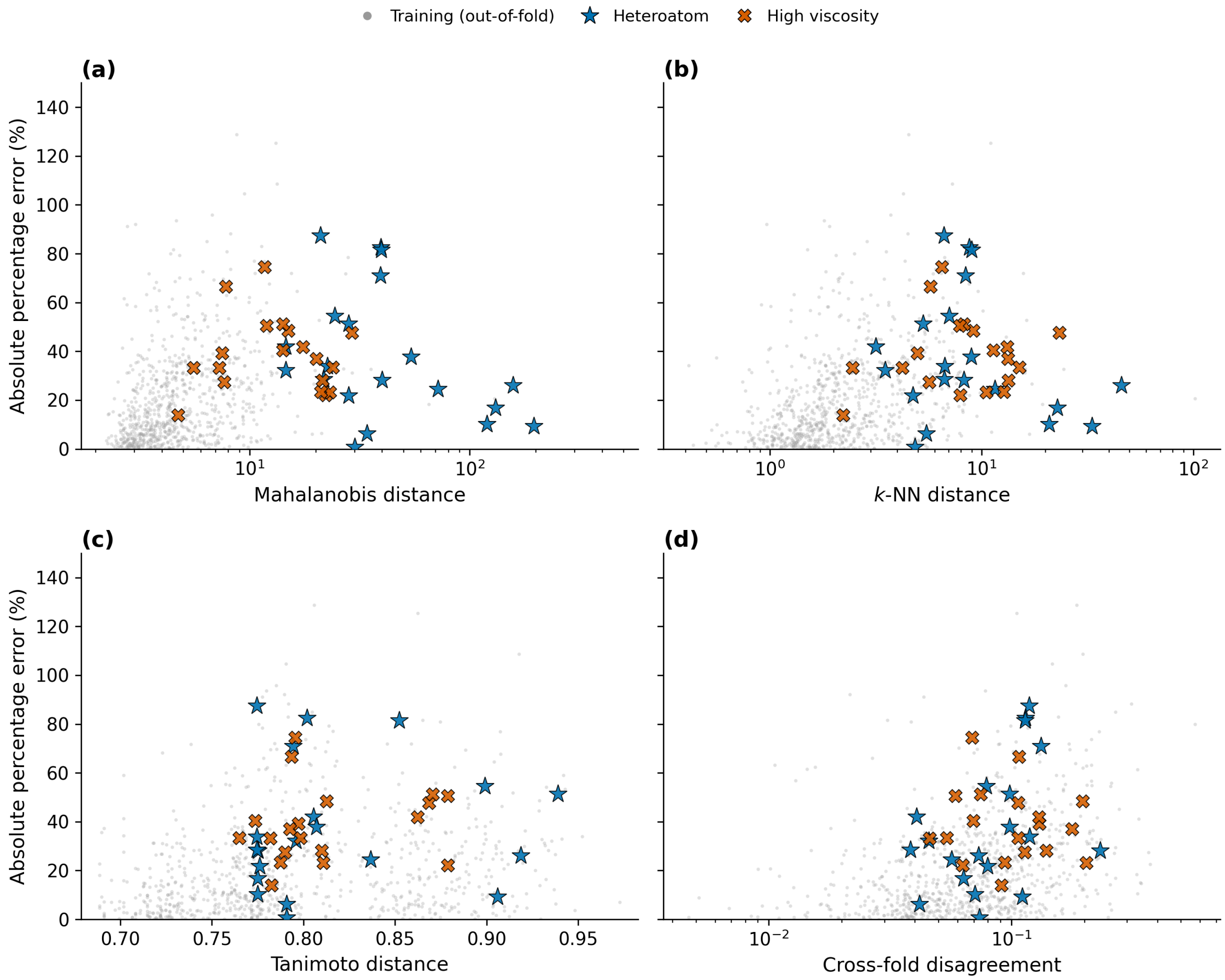

Figure S7: Per-molecule absolute percentage error against each raw signal at 100 °C: (a) Mahalanobis distance, (b) k-NN distance, (c) mean Tanimoto distance, (d) cross-fold disagreement, with conventions as in the main text. The Spearman rank correlation between signal and percentage error is 0.43, 0.44, 0.28 and 0.38 respectively. Unlike at 40 °C, the high-viscosity category no longer dominates the largest errors.

Table S13: Mean absolute percentage error (MAPE) of the pooled training and OOD molecules, split by the combined two-axis flag (Mahalanobis percentile or predicted-viscosity percentile beyond the 90th of the training distribution).

	40 °C		100 °C	
Group	n	MAPE	n	MAPE
In domain (unflagged)	681	24%	686	18%
Flagged OOD	158	42%	158	37%

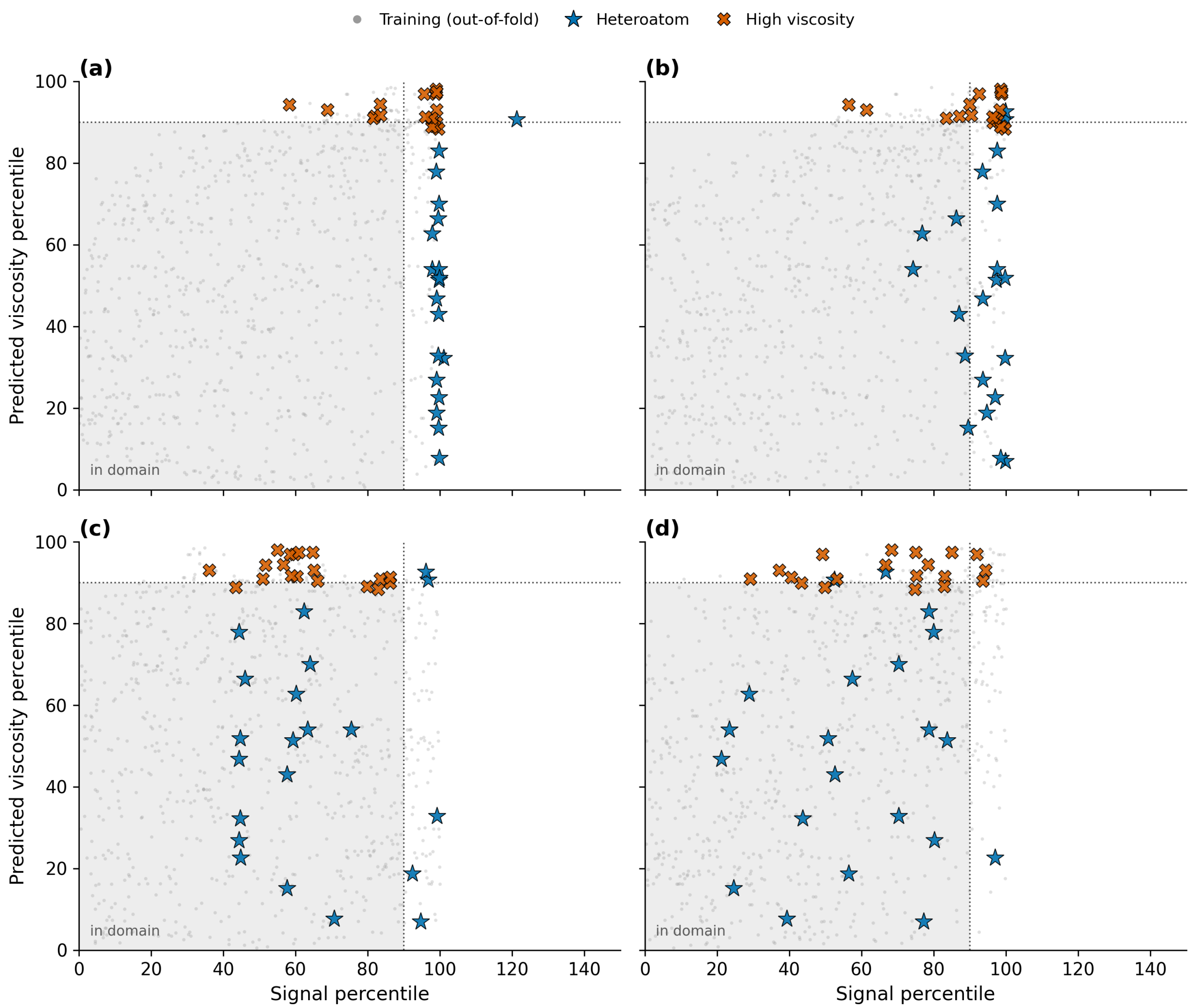

Figure S8: Predicted viscosity, as a percentile of the training labels, against signal percentile at 100 °C for (a) Mahalanobis distance, (b) k-NN distance, (c) Tanimoto distance, and (d) cross-fold disagreement, with conventions as in the main text. The numbers of heteroatom and high-viscosity molecules falling outside that region are 21 of 21 and 19 of 19 in (a), 15 of 21 and 19 of 19 in (b), 5 of 21 and 15 of 19 in (c), and 3 of 21 and 15 of 19 in (d). Two molecules in (a) lie beyond the axis limit and are not shown.

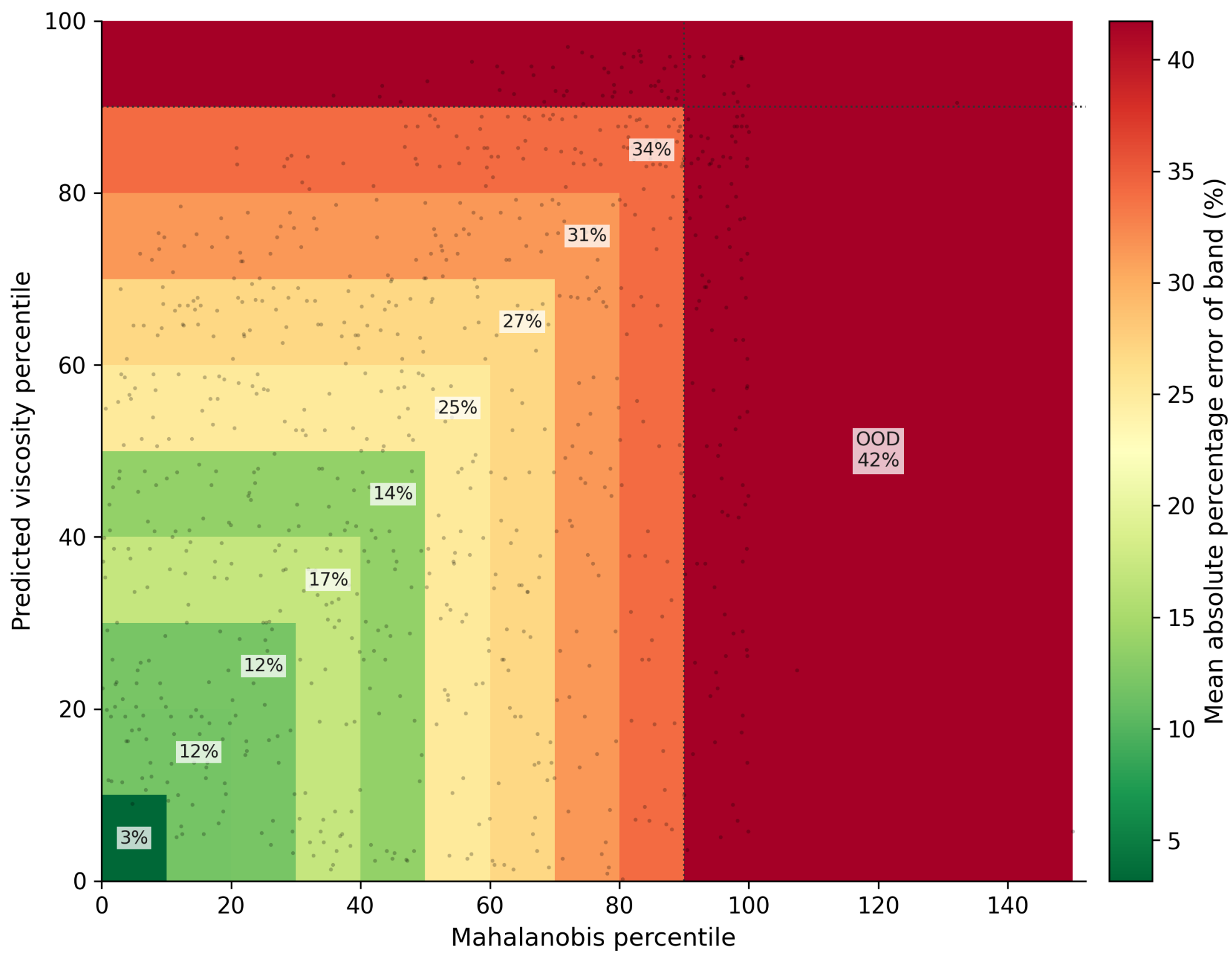

Figure S9: Nested-band view of the two-axis domain at 40 °C. Each L-shaped band pools the training and OOD molecules (gray points) whose larger coordinate (Mahalanobis or predicted-viscosity percentile) falls in that decile, colored by their mean absolute percentage error. The region beyond the 90th percentile on either axis is the flagged OOD region; percentiles beyond 100 use the proportional extension described in the main text, clipped at 150.

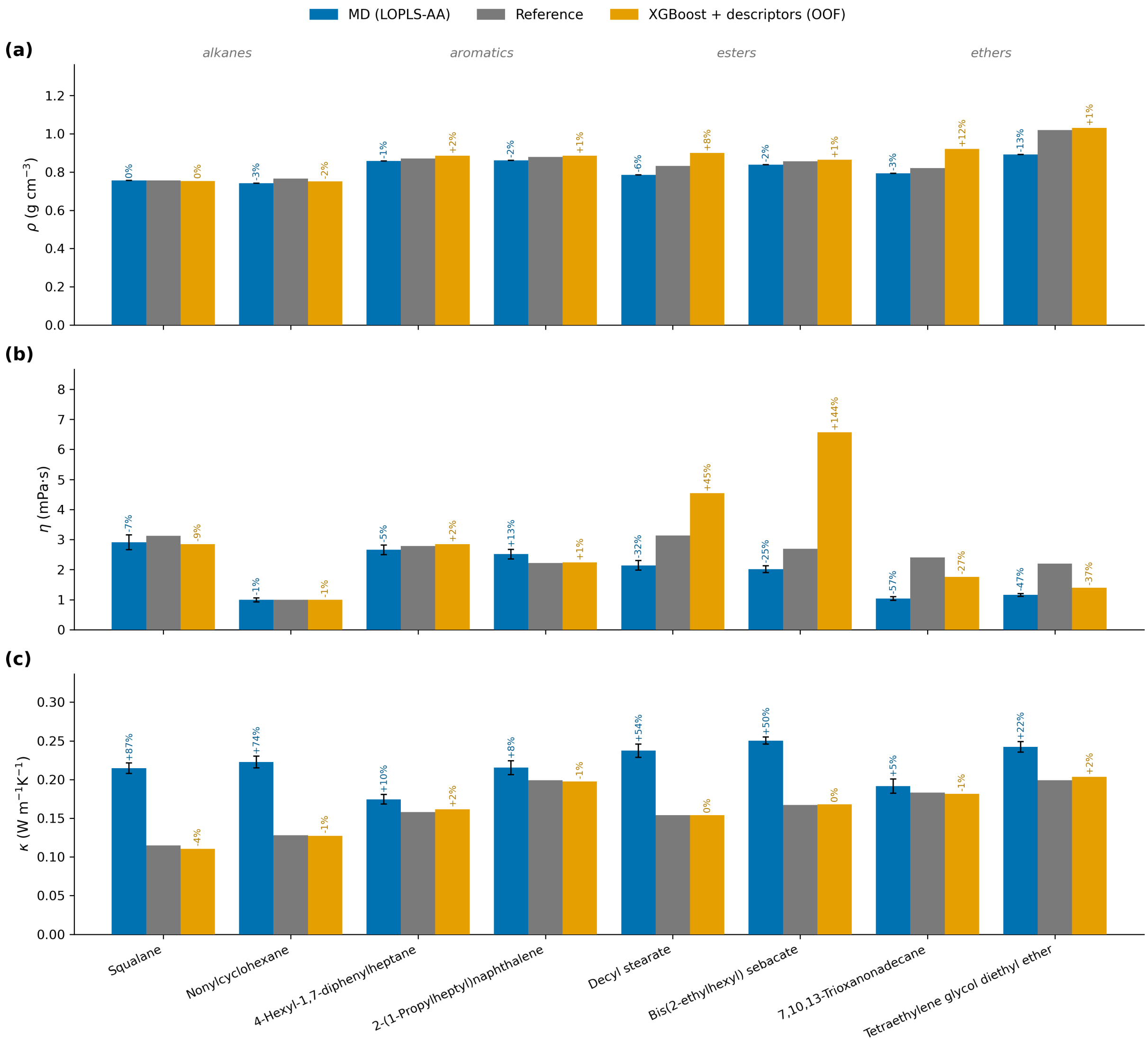

Figure S10: MD (LOPLS-AA, Green–Kubo), reference, and XGBoost descriptor values for the eight target molecules at 100 °C: (a) density, (b) dynamic viscosity, and (c) thermal conductivity, grouped by chemical family. Reference values are obtained by correlation or interpolation from the source databases (see Methods). Labels give the percentage deviation of MD and XGBoost from the reference values. MD error bars are standard deviations over the ten replicas (bootstrap for viscosity). The XGBoost values are OOF predictions, for which the test RMSE is a global rather than per-molecule uncertainty, so no error bars are shown.

Table S14: Per-molecule MD (long-chain optimized all-atom OPLS, LOPLS-AA; Green–Kubo), ML (XGBoost + descriptors, OOF), and reference dynamic viscosity values (mPa·s) for the eight target molecules at 40 and 100 °C. Reference values are obtained by correlation or interpolation from the source databases (see Methods). MD uncertainties are bootstrap standard deviations over the ten replicas.

	40 °C			100 °C		
Molecule	Ref.	MD	ML	Ref.	MD	ML
Squalane	15.06	14.47 ± 1.06	8.73	3.12	2.91 ± 0.25	2.85
Nonylcyclohexane	2.68	2.89 ± 0.17	2.49	1.00	0.99 ± 0.07	0.99
4-Hexyl-1,7-diphenylheptane	15.57	11.71 ± 0.98	26.30	2.79	2.66 ± 0.16	2.85
2-(1-Propylheptyl)naphthalene	13.34	14.92 ± 1.29	11.99	2.22	2.52 ± 0.16	2.24
Decyl stearate	11.18	6.94 ± 0.63	16.08	3.13	2.14 ± 0.16	4.54
Bis(2-ethylhexyl) sebacate	10.45	8.68 ± 0.52	24.81	2.69	2.02 ± 0.12	6.56
7,10,13-Trioxanonadecane	6.40	2.96 ± 0.13	4.83	2.40	1.04 ± 0.06	1.76
Tetraethylene glycol diethyl ether	5.50	4.06 ± 0.17	5.22	2.20	1.16 ± 0.05	1.39

Table S15: Per-molecule MD (LOPLS-AA, Green–Kubo), ML (XGBoost + descriptors, OOF), and reference thermal conductivity values ($W\,m^{-1}\,K^{-1}$) for the eight target molecules at 40 and 100 °C. Reference values are obtained by correlation or interpolation from the source databases (see Methods). MD uncertainties are standard deviations across the ten replicas.

	40 °C			100 °C		
Molecule	Ref.	MD	ML	Ref.	MD	ML
Squalane	0.123	0.241 ± 0.007	0.116	0.115	0.215 ± 0.007	0.111
Nonylcyclohexane	0.136	0.231 ± 0.004	0.136	0.128	0.223 ± 0.007	0.127
4-Hexyl-1,7-diphenylheptane	0.167	0.186 ± 0.009	0.167	0.158	0.174 ± 0.006	0.162
2-(1-Propylheptyl)naphthalene	0.210	0.225 ± 0.009	0.208	0.199	0.215 ± 0.009	0.197
Decyl stearate	0.160	0.251 ± 0.007	0.165	0.154	0.237 ± 0.009	0.154
Bis(2-ethylhexyl) sebacate	0.178	0.248 ± 0.005	0.178	0.167	0.250 ± 0.005	0.168
7,10,13-Trioxanonadecane	0.195	0.211 ± 0.006	0.195	0.183	0.192 ± 0.009	0.182
Tetraethylene glycol diethyl ether	0.212	0.252 ± 0.007	0.228	0.199	0.242 ± 0.007	0.204

Table S16: Per-molecule MD (LOPLS-AA), ML (XGBoost + descriptors, OOF), and reference density values ($g\,cm^{-3}$) for the eight target molecules at 40 and 100 °C. Reference values are obtained by correlation or interpolation from the source databases (see Methods). The reported MD densities are those of the fixed production simulation cell, taken from the final constant-number, constant-pressure, and constant-temperature (NPT) configuration; they agree with the NPT-window means to within 0.3%. Uncertainties are five-block standard deviations of the instantaneous density over the final 0.25 ns of the NPT window.

	40 °C			100 °C		
Molecule	Ref.	MD	ML	Ref.	MD	ML
Squalane	0.796	0.804 ± 0.001	0.806	0.756	0.755 ± 0.003	0.753
Nonylcyclohexane	0.805	0.795 ± 0.001	0.807	0.766	0.742 ± 0.002	0.750
4-Hexyl-1,7-diphenylheptane	0.904	0.902 ± 0.002	0.918	0.870	0.857 ± 0.002	0.886
2-(1-Propylheptyl)naphthalene	0.914	0.907 ± 0.002	0.924	0.879	0.861 ± 0.002	0.885
Decyl stearate	0.863	0.834 ± 0.002	0.904	0.832	0.785 ± 0.002	0.899
Bis(2-ethylhexyl) sebacate	0.899	0.891 ± 0.003	0.919	0.857	0.839 ± 0.002	0.864
7,10,13-Trioxanonadecane	0.859	0.853 ± 0.002	0.959	0.820	0.794 ± 0.002	0.920
Tetraethylene glycol diethyl ether	1.069	0.959 ± 0.002	1.100	1.019	0.891 ± 0.003	1.031

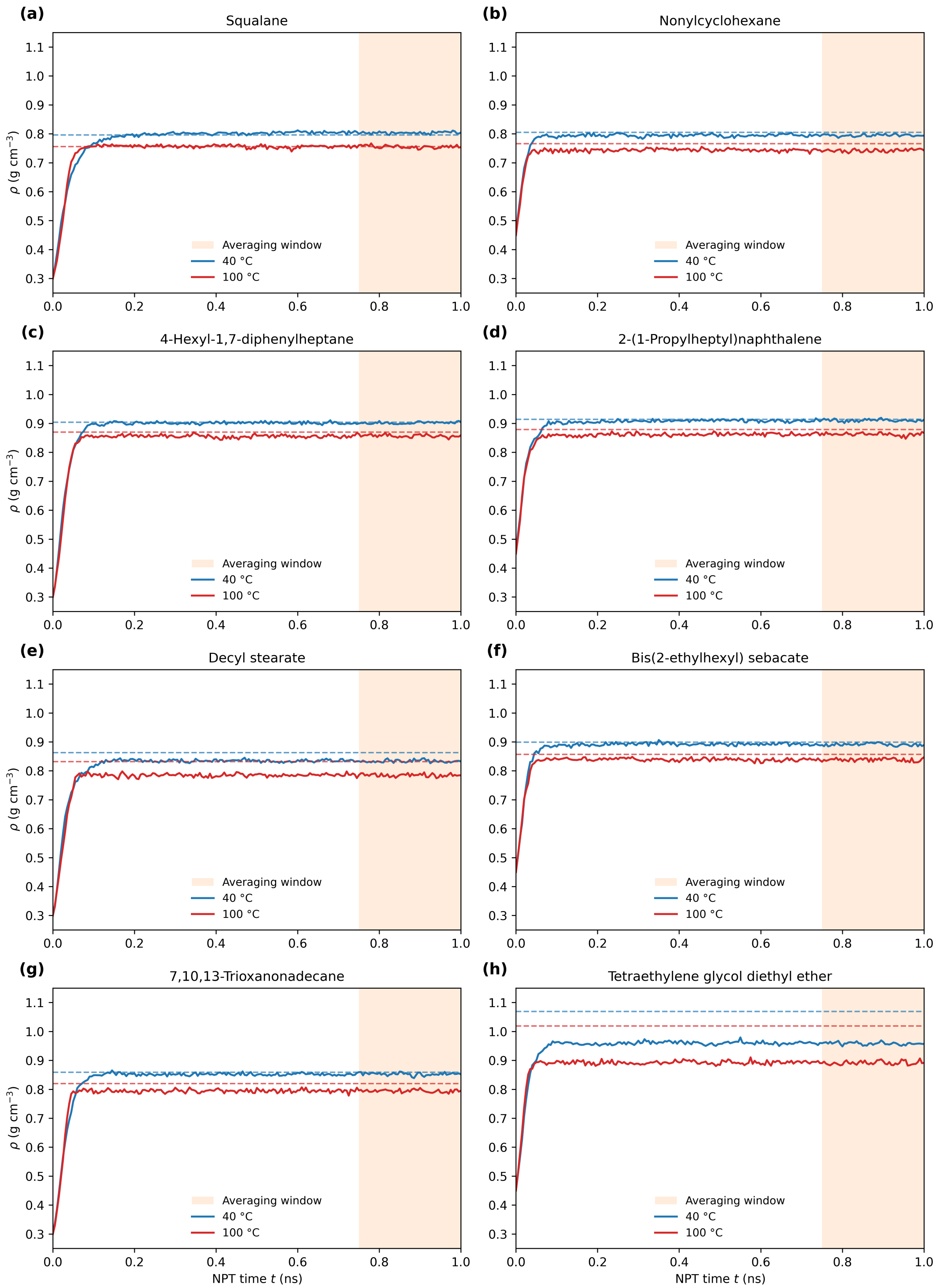

Figure S11: Instantaneous mass density during the 1 ns NPT equilibration at 40 °C (blue) and 100 °C (red) for (a) squalane, (b) nonylcyclohexane, (c) 4-hexyl-1,7-diphenylheptane, (d) 2-(1-propylheptyl)naphthalene, (e) decyl stearate, (f) bis(2-ethylhexyl) sebacate, (g) 7,10,13-trioxanonadecane, and (h) tetraethylene glycol diethyl ether. Dashed lines are the reference densities and the shaded region is the final 0.25 ns averaging window.

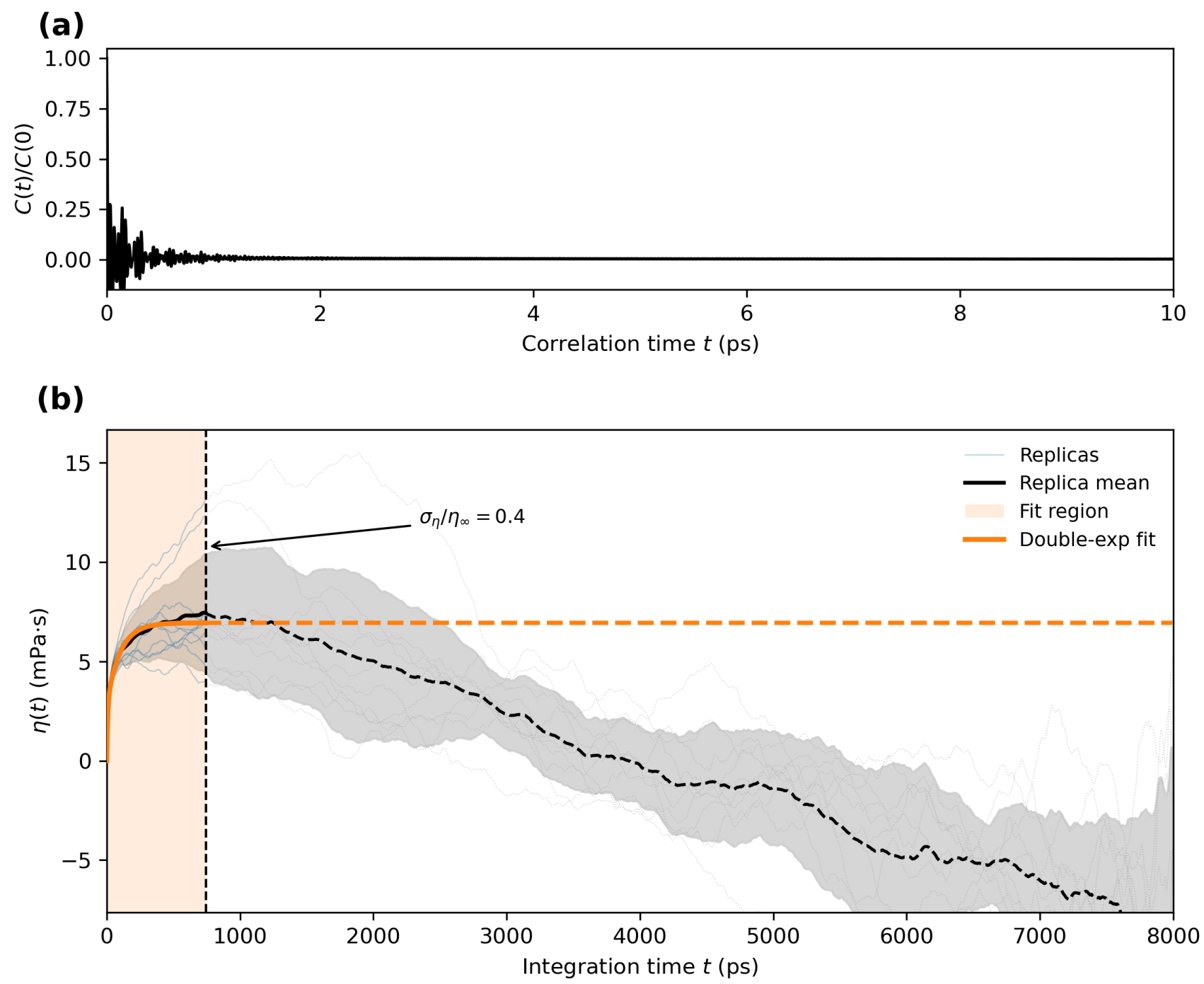

Figure S12: (a) Stress autocorrelation function and (b) running Green–Kubo viscosity integral $\eta(t)$ for decyl stearate at 40 °C (ten 8 ns replicas), with the double-exponential time-decomposition method (TDM) fit taken up to the $\sigma_\eta/\eta_\infty = 0.4$ cutoff. Blue curves are individual replicas, the black curve the replica mean, and the shaded band ±1 standard deviation.

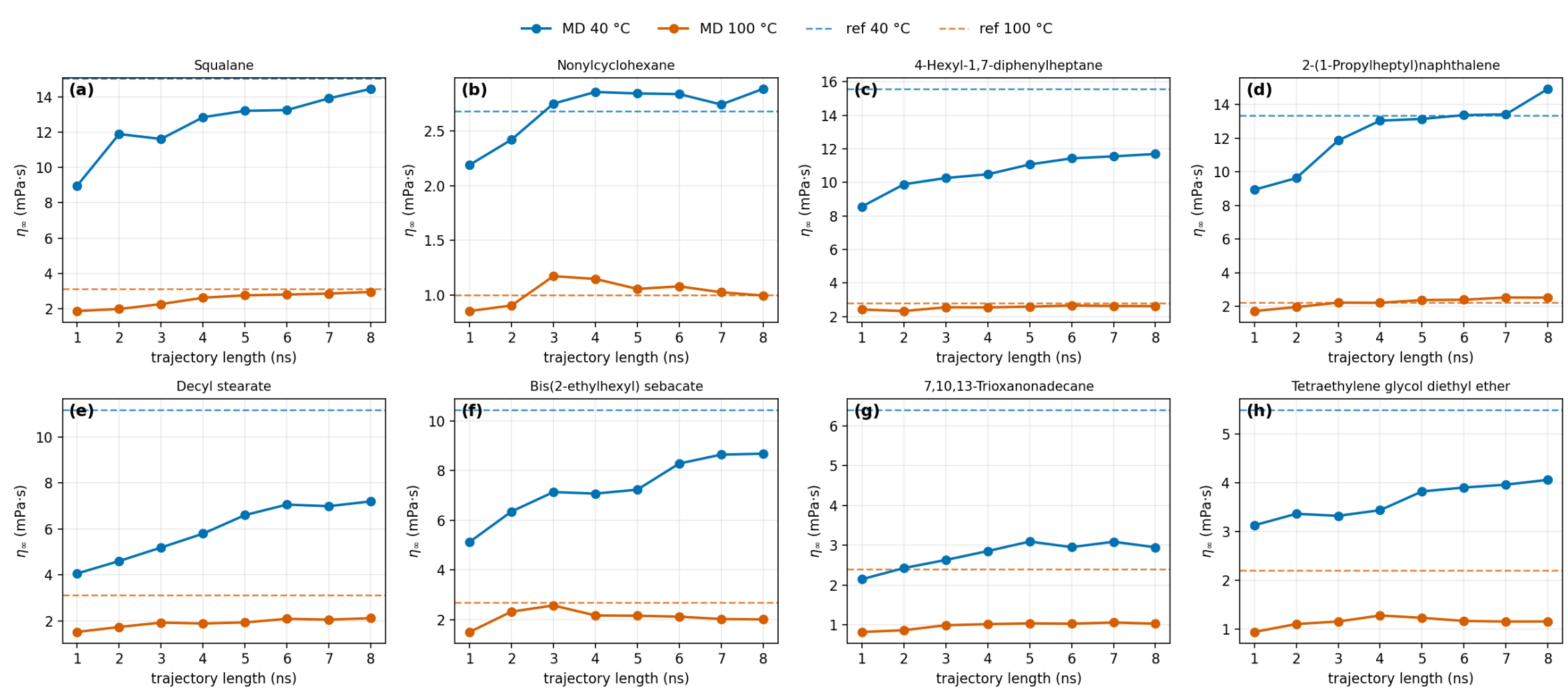

Figure S13: Converged Green–Kubo viscosity η_∞ against total trajectory length for the eight target molecules (a–h, ten 8 ns replicas) at 40 °C (blue) and 100 °C (red); dashed lines are the reference values. For every molecule η_∞ rises well beyond 2 ns and approaches a plateau by 6–8 ns.

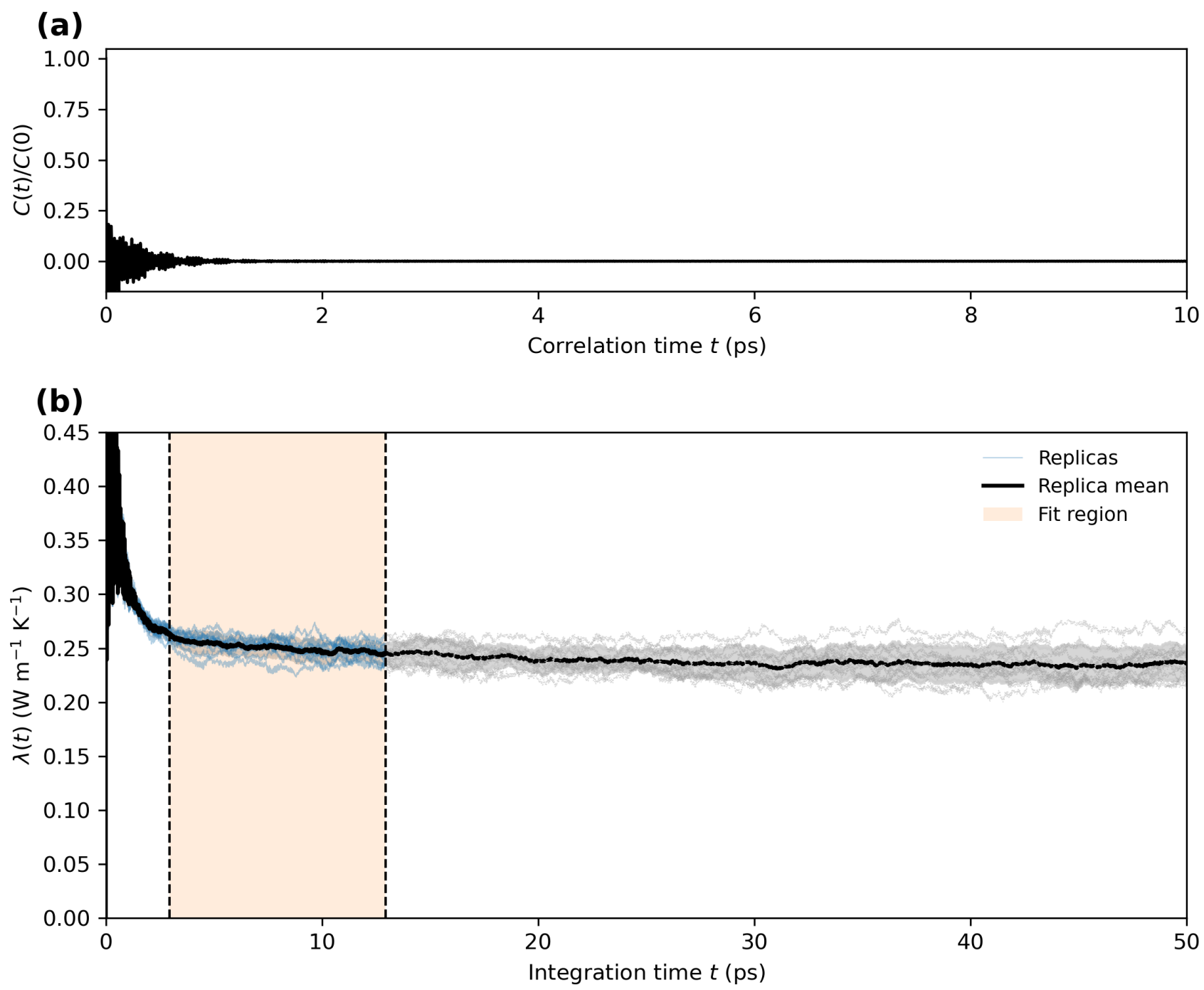

Figure S14: (a) Heat current autocorrelation function and (b) running Green–Kubo thermal conductivity integral $\kappa(t)$ for decyl stearate at 40 °C (ten 8 ns replicas); κ is read over the shaded plateau window. Blue curves are individual replicas, the black curve the replica mean, and the shaded band ±1 standard deviation.

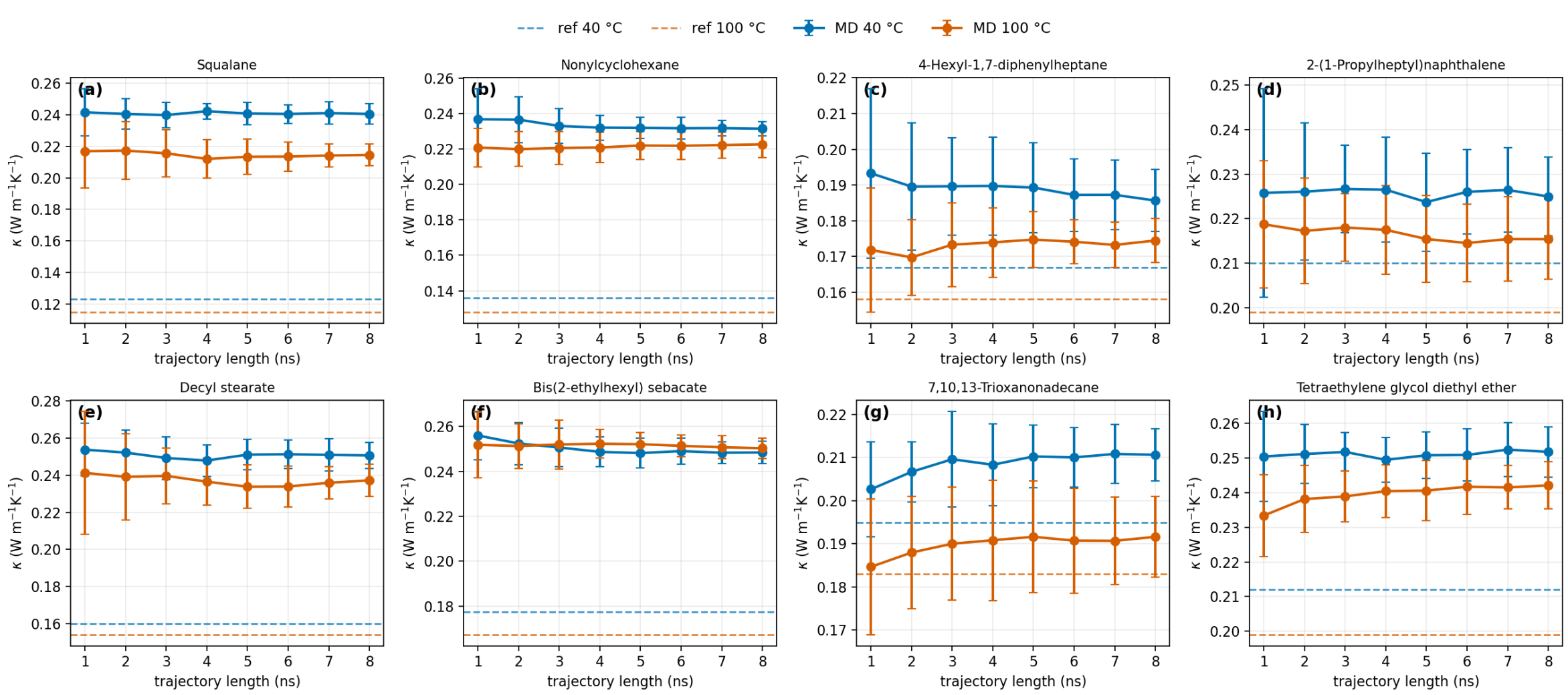

Figure S15: Green–Kubo thermal conductivity κ against total trajectory length for the eight target molecules (a–h), read from the plateau window of the running integral, at 40 °C (blue) and 100 °C (red). Error bars are standard deviations across the ten replicas and dashed lines are the reference values. Unlike the viscosity, κ is converged within 1–2 ns.

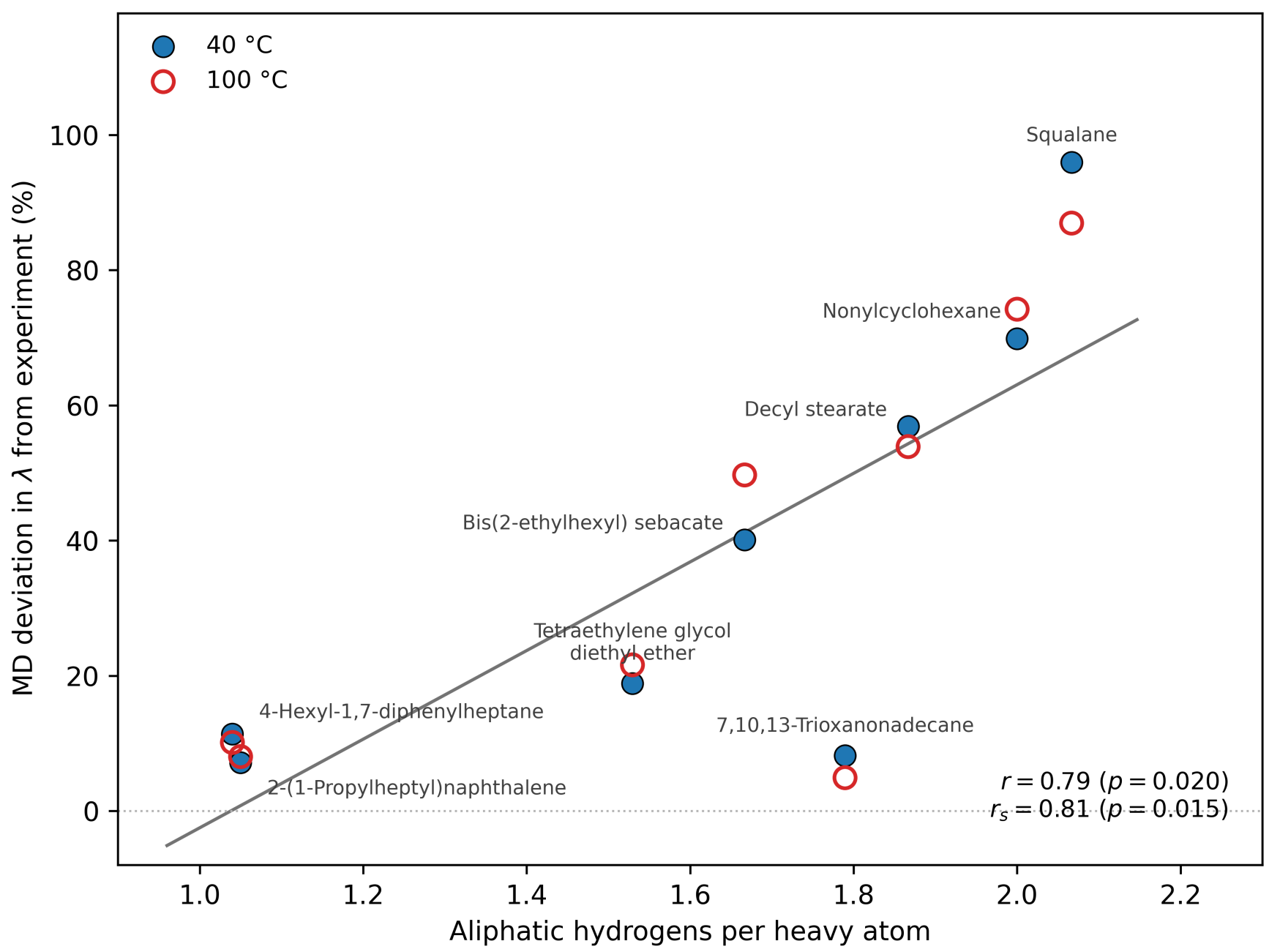

Figure S16: Percentage deviation of the MD thermal conductivity from the reference values against the number of aliphatic hydrogens per heavy atom for the eight target molecules, at 40 °C (filled blue) and 100 °C (open red); the gray line is a least-squares fit to the 40 °C values. The two polyethers fall below the fitted line.

References

(1) Huber, M. L.; Lemmon, E. W.; Bell, I. H.; McLinden, M. O. The NIST REFPROP Database for Highly Accurate Properties of Industrially Important Fluids. *Industrial and Engineering Chemistry Research* **2022**, *61*, 15449–15472.

(2) Mammone, A.; Turchi, M.; Cristianini, N. Support vector machines. *Wiley Interdisciplinary Reviews: Computational Statistics* **2009**, *1*, 283–289.

(3) Cortes, C.; Vapnik, V.; Saitta, L. Support-Vector Networks. *Machine Leaming* **1995**, *20*, 273–297.

(4) Breiman, L. Random Forests. *Machine Learning* **2001**, *45*, 5–32.

(5) Cover, T.; Hart, P. Nearest neighbor pattern classification. *IEEE Transactions on Information Theory* **1967**, *13*, 21–27.

(6) Babajide Mustapha, I.; Saeed, F. Bioactive molecule prediction using extreme gradient boosting. *Molecules* **2016**, *21*, 983.

(7) Dong, J.; Peng, L.; Yang, X.; Zhang, Z.; Zhang, P. XGBoost-based intelligence yield prediction and reaction factors analysis of amination reaction. *Journal of Computational Chemistry* **2022**, *43*, 289–302.

(8) Jiang, D.; Wu, Z.; Hsieh, C.-Y.; Chen, G.; Liao, B.; Wang, Z.; Shen, C.; Cao, D.; Wu, J.; Hou, T. Could graph neural networks learn better molecular representation for drug discovery? A comparison study of descriptor-based and graph-based models. *Journal of Cheminformatics* **2021**, *13*, 1–23.

(9) Chew, A. K.; Sender, M.; Kaplan, Z.; Chandrasekaran, A.; Elk, J. C.; Browning, A. R.; Kwak, H. S.; Halls, M. D.; Afzal, M. A. F. Advancing material property prediction: using physics-informed machine learning models for viscosity. *Journal of Cheminformatics* **2024**, *16*.

(10) Chen, T.; Guestrin, C. Xgboost: A scalable tree boosting system. Proceedings of the 22nd acm sigkdd international conference on knowledge discovery and data mining. 2016; pp 785–794.

(11) Tang, M.; Li, B.; Chen, H. Application of message passing neural networks for molecular property prediction. *Current Opinion in Structural Biology* **2023**, *81*, 102616.

(12) Bilodeau, C.; Kazakov, A.; Mukhopadhyay, S.; Emerson, J.; Kalantar, T.; Muzny, C.; Jensen, K. Machine learning for predicting the viscosity of binary liquid mixtures. *Chemical Engineering Journal* **2023**, *464*, 142454.

(13) Chew, A. K.; Sender, M.; Kaplan, Z.; Chandrasekaran, A.; Elk, J. C.; Browning, A. R.; Kwak, H. S.; Halls, M. D.; Afzal, M. A. F. Advancing material property prediction: using physics-informed machine learning models for viscosity. *Journal of Cheminformatics* **2024**, *16*, 31.

(14) Yang, K.; Swanson, K.; Jin, W.; Coley, C.; Eiden, P.; Gao, H.; Guzman-Perez, A.; Hopper, T.; Kelley, B.; Mathea, M.; Palmer, A.; Settels, V.; Jaakkola, T.; Jensen, K.; Barzilay, R. Analyzing Learned Molecular Representations for Property Prediction. *Journal of Chemical Information and Modeling* **2019**, *59*, 3370–3388.

(15) Veličković, P.; Cucurull, G.; Casanova, A.; Romero, A.; Lio, P.; Bengio, Y. Graph attention networks. *ArXiv Preprint arXiv:1710.10903* **2017**,

(16) Li, Z.; Liu, F.; Yang, W.; Peng, S.; Zhou, J. A Survey of Convolutional Neural Networks: Analysis, Applications, and Prospects. *IEEE Transactions on Neural Networks and Learning Systems* **2022**, *33*, 6999–7019.

(17) Li, Z.; Jiang, M.; Wang, S.; Zhang, S. Deep learning methods for molecular representation and property prediction. *Drug Discovery Today* **2022**, *27*, 103373.

(18) Kimber, T. B.; Gagnebin, M.; Volkamer, A. Maxsmi: maximizing molecular property prediction performance with confidence estimation using smiles augmentation and deep learning. *Artificial Intelligence in the Life Sciences* **2021**, *1*, 100014.

(19) Wei, J. N.; Duvenaud, D.; Aspuru-Guzik, A. Neural networks for the prediction of organic chemistry reactions. *ACS Central Science* **2016**, *2*, 725–732.

(20) Çamur, H.; Al-Ani, A. M. R. Prediction of oxidation stability of biodiesel derived from waste and refined vegetable oils by statistical approaches. *Energies* **2022**, *15*, 407.

(21) Olivecrona, M.; Blaschke, T.; Engkvist, O.; Chen, H. Molecular de-novo design through deep reinforcement learning. *Journal of cheminformatics* **2017**, *9*, 1–14.

(22) Rumelhart, D. E.; Hinton, G. E.; Williams, R. J. Learning representations by back-propagating errors. *nature* **1986**, *323*, 533–536.

(23) Ruder, S. An overview of gradient descent optimization algorithms. *ArXiv Preprint arXiv:1609.04747* **2016**,

(24) Vaswani, A.; Shazeer, N.; Parmar, N.; Uszkoreit, J.; Jones, L.; Gomez, A. N.; Kaiser, Ł.; Polosukhin, I. Attention is all you need. 31st Conference on Neural Information Processing Systems. 2017; p 5998–6008.

(25) Hochreiter, S. The vanishing gradient problem during learning recurrent neural nets and problem solutions. *International Journal of Uncertainty, Fuzziness and Knowledge-Based Systems* **1998**, *6*, 107–116.

(26) Chithrananda, S.; Grand, G.; Ramsundar, B. ChemBERTa: large-scale self-supervised pretraining for molecular property prediction. *ArXiv Preprint arXiv:2010.09885* **2020**,

(27) Ahmad, W.; Simon, E.; Chithrananda, S.; Grand, G.; Ramsundar, B. Chemberta-2: Towards chemical foundation models. *ArXiv Preprint arXiv:2209.01712* **2022**,

(28) Singh, R. et al. ChemBERTa-3: An Open Source Training Framework for Chemical Foundation Models. *Digital Discovery* **2026**, *5*, 662.

(29) Zhou, R.; Bao, L.; Bu, W.; Zhou, F. Exploring high-performance viscosity index improver polymers via high-throughput molecular dynamics and explainable AI. *npj Computational Materials* **2025**, *11*, 52.

(30) Maćkiewicz, A.; Ratajczak, W. Principal components analysis (PCA). *Computers & Geosciences* **1993**, *19*, 303–342.

(31) McInnes, L.; Healy, J.; Melville, J. Umap: Uniform manifold approximation and projection for dimension reduction. *ArXiv Preprint arXiv:1802.03426* **2018**,

(32) Rudnick, L. R. *Synthetics, mineral oils, and bio-based lubricants: chemistry and technology*; CRC press, 2020.
